# FlowerSec: An AI-Enhanced Security Framework for Clustered Wireless Sensor Networks

**DOI:** 10.3390/s26206530

**Published:** 2026-10-15

**Authors:** Ahmed Alhomoud, Adel R. Alharbi, Najeeb Ahmed Altorkmani

**Affiliations:** 1 Department of Computer Science, College of Science, Northern Border University, Arar 91431, Saudi Arabia; aalhomoud@nbu.edu.sa; 2 College of Computing and Information Technology, University of Tabuk, Tabuk 71491, Saudi Arabia; 441010946@st.ut.edu.sa

**Keywords:** wireless sensor networks, intrusion detection, machine learning, clustering, SHAP, network security, fault tolerance, energy efficiency

## Abstract

Existing clustered wireless sensor network (WSN) security schemes treat intrusion detection as an add-on and rely on supervised models that fail against unseen attacks. We propose FlowerSec, an intrusion detection framework that uses the clustering topology itself as the security primitive for clustered WSNs. Sensors are organized into “flowers” comprising a seed (cluster head) and up to four leaves within a trust boundary. Each seed runs a dual-engine intrusion detection system: a SHAP-optimized LightGBM for known attacks and an unsupervised AutoEncoder for zero-day threats, connected by a confidence-gated escalation protocol. On the WSN-DS benchmark, the lightweight engine achieves a Macro F1-score of 0.9494 using eight features at 0.0066 ms and 1.74 microjoules per packet. The unsupervised engine detects 96.4% of unseen flooding attacks, whereas the supervised engine achieves 0.8% recall. Tiered activation of the two engines extends network lifetime by 121 days over flat architectures.

## 1. Introduction

Wireless Sensor Networks (WSNs) are deployed in environmental monitoring, healthcare, military surveillance, and smart infrastructure. However, their operational life is constrained by limited battery capacity and modest onboard processing. At the same time, the shared wireless medium exposes nodes to compromise. An attacker who captures or impersonates a node can alter data, disrupt routing links, and exhaust remaining battery reserves.

These constraints make security and energy management closely linked. In clustered networks, traffic-centric attacks including blackhole, grayhole, flooding, and scheduling exploits threaten network reliability. By selectively dropping packets or introducing transmission delays, compromised nodes degrade throughput while forcing neighboring nodes to waste energy on retransmissions. Consequently, defense mechanisms for WSNs must preserve confidentiality, authenticity, and availability without exceeding the hardware’s strict processing and power limits.

One prevalent strategy for addressing energy consumption is through clustering. This methodology involves aggregating several nodes to form a “cluster”, wherein one node is designated as the master to facilitate communication with the master nodes of other clusters. By centralizing the transmission of messages through a single node, this approach minimizes redundancy in communication and alleviates the overall burden on the network.

The LEACH protocol exemplifies how such a clustering framework can contribute to energy efficiency while concurrently presenting attractive targets for system vulnerabilities. Given that all nodes rely on the integrity of their cluster head, recent advancements have sought to incorporate both security measures and fault tolerance into energy-aware clustering frameworks. However, these enhancements are often implemented opportunistically rather than being designed into the system from the outset.

In this paper, we introduce FlowerSec, an innovative security framework tailored for clustered wireless sensor networks (WSNs), driven by our artificial intelligence methodology. This framework integrates the fault-tolerant flower architecture with a lightweight mechanism for identifying components of WSN sensors and actuators. Such an architecture inherently establishes security boundaries while facilitating intelligent power management through the identification of sensor components within the network that require specific quality-of-service (QoS) levels. FlowerSec provides three hierarchical levels with their QoS and security phases for different types of applications as follows:

- Layer 1 for mission critical applications (high security, QoS guarantees). The components could include things like heart monitoring units or sensors associated with mission-critical applications that demand guaranteed uptime and data integrity.
- Layer 2 for normal daily monitoring which could adapt when there is need or on call (moderate security and smart QoS). These components could include things like surveillance cameras, door-open sensors, etc.
- Layer 3 for legacy statistics logging, thus supporting a variety of hardware (low security with best effort QoS). These components could include humidity sensors, light sensors, etc.

Meanwhile, in practical deployments, the layer assignment is determined by the application and the sensor’s operational role. For example, mission-critical sensors found in structural health monitors or medical devices are part of layer 1 at deployment time, based on their sensing function. Regular monitoring sensors like environmental sensors and door sensors are assigned to layer 2, whilst layer 3 has background logging sensors assigned to it like temperature loggers. This assignment is performed during network installation by the base station based on the application profile which can be dynamically reconfigured if a sensor’s operational priority changes.

In terms of controlling the devices of each layer while observing security and faults related to the IoT devices, FlowerSec is equipped with a new mechanism which can learn and anticipate the type of IoT device it may encounter, given the type of work to be performed. Thus, FlowerSec develops in each of the devices associated with its security layers a lightweight ML scheme which identifies the components whose behavior adapts according to operational priorities. FlowerSec’s ML agents have three forms:

1. Active ML, where ML is always on, e.g., for level-1 (high-security) devices. These sensors, such as medical monitoring equipment, require high availability and reliability.
2. Sleep ML, where ML is idle.
3. Alert/on call ML, where its functionality activates only when its sensing devices are needed and deactivates soon afterwards to preserve energy, e.g., level-2 and -3 devices.

When a flower in a particular layer experiences failures (i.e., a type I Flower), it must regenerate using one of three mechanisms provided by the FlowerSec framework to allow the entire community to recover: single-layer regeneration through flower expansion, multilayered regeneration mechanisms where the failed layers are recovered by other layers in the flower that are more secure than them, and a third hybrid option to combine these two regeneration mechanisms. These regeneration mechanisms assist in allowing the flower or its layers to degrade gracefully whenever subjected to malicious actions within specified energy thresholds, given as the data loss percentage against the flower node depletion. The hybrid approach provides better degradation performance compared to its counterpart mechanisms during random malicious attacks. These regeneration mechanisms effectively support the longevity and sustainability of the community within the specified service level, i.e., 99% against different malicious attacks.

The main contributions of this work are summarized as follows:

1. Flower Topology: A novel hierarchical WSN architecture where a central seed node is surrounded by up to four leaf nodes, naturally defining security boundaries and enabling energy-efficient tiered sleep scheduling across three QoS layers.
2. Dual-Engine IDS: A lightweight supervised LightGBM detector optimized via SHAP feature selection to use only eight features, complemented by an unsupervised AutoEncoder for zero-day threat detection through a confidence-gated escalation protocol (Algorithm 1).
3. Tiered ML Activation: A multilayer strategy (layer 1: always-on; layer 2: on-demand; layer 3: sleeping) that extends network lifetime by 121 days compared to flat architectures.
4. Recovery Mechanisms: Three fault tolerance strategies (single-layer expansion, multilayer cross-support, and hybrid recovery) with ML-predicted self-healing for proactive failure avoidance.
5. Comprehensive Validation: Evaluation across four datasets (WSN-DS, SensorNetGuard, NSL-KDD, CIC-IDS2017) with 10-fold CV, LOOCV, zero-day leave-one-attack-out simulation, adversarial evasion analysis, noise robustness testing, and federated learning simulation.

**Algorithm 1** Confidence-gated dual-engine cascade at the seed node**Require:** Incoming packet feature vector *x*, supervised model $M_{sup}$, AutoEncoder $M_{ae}$, confidence threshold τ=0.90, reconstruction threshold θ
**Ensure:** Final verdict: *Normal* or *Malicious*
1:$p,c\leftarrow M_{sup}.$predict_proba(*x*)      ▹ Get class prediction and max confidence2:**if** c≥τ **then**3:     **return** *p*             ▹ High confidence: trust Engine 1 immediately4:**else**5:     **if** p==Normal **then**6:          $e\leftarrow M_{ae}.$reconstruction_error(*x*)    ▹ Low-confidence Normal: wake Engine 27:          **if** e>θ **then**8:               **return** *Malicious*          ▹ AutoEncoder flags zero-day anomaly9:          **else**10:             **return** *Normal*11:        **end if**12:    **else**13:        **return** *p*             ▹ Low-confidence attack: trust Engine 1 alert14:    **end if**15:**end if**

The remainder of this paper is structured as follows: Section 2 evaluates related work on the system to gain knowledge about WSN clustering, security, and packets. Section 3 contains information about the flower architecture, which includes the basic building blocks and the multiple layer structure. Section 4 contains the recovery mechanisms of the framework. Section 5 introduces the AI-enhanced security framework and the machine learning methodology. Section 6 details the experimental validation and discusses the results. Finally, Section 7 concludes the paper and outlines directions for future work.

## 2. Related Work

Clustering, routing, energy management, and security are the core areas of research in wireless sensor networks. The key literature review from each of these areas, along with the positioning of the FlowerSec framework, is presented in this section.

Clustering has remained a major approach for improving network scalability and energy efficiency of wireless sensor networks [1]; the random rotation of a cluster head was first proposed in LEACH for equally distributing energy among all networks and was further refined by various protocols like HEED and PEGASIS by adding support for energy-aware clusters and chain-based data aggregation methods [2,3]. Recently, CREN-RCL was implemented by assigning energy-efficient clusters for sensor nodes based on their distance and communication to their neighbors nodes; that method could achieve a classification accuracy of up to 94.38% with a forwarding ratio of 0.982 [4].

Another approach using fuzzy inference clustering for energy-efficient routing employs Mamdani-type fuzzy inference systems for adaptive cluster formation. It ensures balanced and stable clustering through cluster head optimization based on the inspired Dynamic Adaptive Fig Tree Wasp Sum Biotic Coevolutionary Optimization [5] algorithm, which ensures low energy consumption of 1.02 J with a good throughput of 0.93 Mbps, highlighting the potential of bio-inspired optimization techniques for WSNs [5].

Securing networks has become a top priority, especially since resource-limited sensor nodes are highly vulnerable to threats. Attacks can compromise data integrity, disrupt communication, and rapidly deplete battery life, risking complete network failure. Consequently, researchers are turning to machine learning to create models that identify normal traffic patterns and quickly detect unusual or harmful activities.

A range of surveys of anomaly intrusion detection systems have been conducted [6]. To test the network defenses, the WSN DS dataset is the main standard, with over than 374,000 real-world examples of both safe traffic and actual attacks that were collected for use in experiments by survey researchers [7]. Such surveys use a number of classifiers including voting classifiers combining Random Forest, K-Nearest Neighbors and XGBoost. When combining Particle Swarm Optimization, the accuracy reaches 99.99% [8]. When using XGBoost in a lightweight approach, an accuracy near 99.73% can also be achieved with a concurrent 68% time reduction in processing rate compared to other classifiers. This can help with the constrained resource use in WSNs [9].

WSN research uses the “Flower” concept mainly for optimization algorithms. SEEF (Secured Energy-Efficient Framework) proposes a cross-layer design using flower pollination optimization for dynamic optimal routing and achieves a throughput of 0.57 MB/s and a packet delivery ratio of 96% [10]. Such a design relies on Sybil attack detection at the cluster-head and base-station levels, and trust values are also computed based on residual energy and rated dropping. Federating disjoint segments (algorithm FLOWER): this method groups the disjoint segments forming clusters, which forms an intercluster star topology where each cluster is appointed using one distinct mobile agent. Although they share the same word, the core working of those systems is different from our proposed FlowerSec. Currently, research utilizing the “flower” concept primarily focuses on either flower pollination as an optimization metaheuristic, network federation as an optimization metaheuristic, or on networking to connect with other small networks to act together (federate). FlowerSec was inspired by that concept, introducing a new physical topology for organizing sensors. In our proposed topology, a flower consists of a central seed node surrounded by up to four leaf nodes; however, at each flower-unit (cluster), there are natural security boundaries making it possible for an AI-powered intrusion detection system to detect malicious activity using machine learning concepts; then, with the help of communication between the flower’s “seed” and neighboring flower “seeds” (clusters), it becomes possible to identify the adversary’s location and take all possible steps to eliminate the danger (with the help of Drone Swarm and/or the help from an ally network or the control room) [11].

Recent AI-based WSN security approaches are compared in Table 1.

In addition, capacity and spectrum-aware communication frameworks for WSN-based smart grid applications [12] optimize throughput and spectrum efficiency but treat the network as trusted; they provide no intrusion detection layer and no recovery mechanism, leaving them exposed to traffic-oriented attacks such as blackhole, grayhole, and flooding. FlowerSec complements such communication-layer designs by embedding detection and recovery inside the topology itself.

Even with significant advancements in clustering, intrusion detection and nature-inspired (flower) optimization, research lacks an integrated framework that leverages both structured nature-inspired topology and AI-empowered security features in WSNs. Most approaches lean towards clustering, which is effective in terms of energy consumption and scalability but lacks a built-in security boundary or fault security. Thus, the WSN-DS dataset is extensively used for intrusion detection in this unique architecture called flower base. The FlowerSec solution addresses this research gap by utilizing the first-ever integrated approach implementing flower topology and AI-enhanced security and trying it on a standard dataset for a real-time perspective rather than relying purely on a complex network.

## 3. Flower Architecture

The basis of the FlowerSec solution is the flower architecture. The following describes the architecture components, the structure they form and general rules of their interactions in the solution.

### 3.1. Basic Building Block: The Flower

FlowerSec is based entirely around the flower topology, a self-sustained cluster built around one strong node (seed), which acts as the CH. Up to 4 leaves can exist orbiting the seed inside the trust zone, the leaves being the nodes collecting the data.

**Figure 1:**
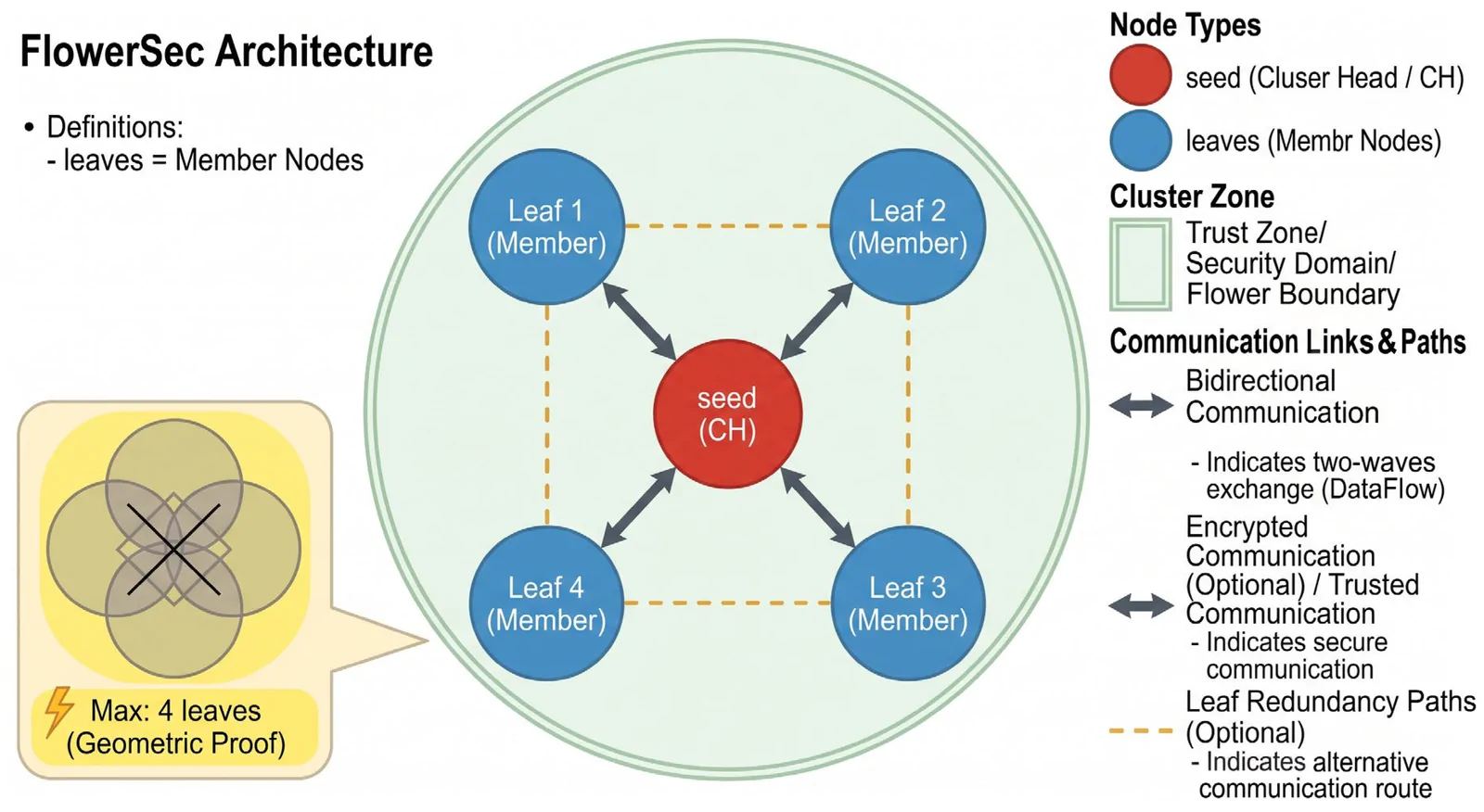
FlowerSec architecture—basic building block. The flower organizes sensor nodes into a central seed (cluster head) and up to four leaf member nodes. The trust zone/security domain defines the security perimeter, with bidirectional communication links enabling data reporting and command dissemination.

The four-leaf limit follows from two explicit constraints rather than from congruent circles kissing. Let *r* denote the leaf sensing radius, *R* the reliable communication radius of the seed, and $d_{l}$ the distance between two adjacent leaf centers. Leaves must not overlap, so $d_{l}\geq 2r$, and every leaf center must lie within the seed’s reliable radio range. Placing *k* leaf centers on the circle of radius *R*, the chord between adjacent centers is 2R sin(π/k), and the non-overlap condition yields k≤π/arcsin(r/R). For R=2r, that bound recovers the classical kissing number of six; with the link-budget margin used in our simulations, R=1.5r, it evaluates to 4.30, i.e., at most four leaves. The limit of four is therefore a consequence of the communication-range constraint, not of pure packing. Two adjacent leaves must still satisfy the non-overlap condition $d_{l}\geq 2r$, where *r* is the sensing radius at any given time, to ensure the leaves do not overlap but still maintain full coverage as seen in Figure 1, while avoiding as much loss of coverage as possible during rotation.

Establishing that, in effect, marks and divides the territory within which the seed can keep watch and authenticate its members, leaving it a small and localized area to apply its security policies. These connections are fundamental bidirectional communication links. This facilitates the continuous flow of sensor information from leaves to be aggregated on the seed and information like configuration updates (or security settings) to be disseminated by the seed to its leaves.

Further supporting this line, the architecture provides protection for communication exchanges as it allows opt-in encrypted communication, suggesting that data privacy, while not mandatory in this respect, represents a more trusted method of communication in this case. The more generalized method of trusted communication may be relied upon in these operations within the architecture where applicable. Another possible add-on feature to the present design is an optional leaf redundancy path, which works without the intervention of a seed and allows communication to continue as usual along another alternative route. This allows the cluster to have an inbuilt defense against being isolated from a node or a network link, allowing the FlowerSec architecture to cope with such problems, while also making it robust enough to handle small faults and cope with some self-healing within the cluster in some locations. The seed in this state not only serves as data aggregator for further collection, but a strong local security administrator that enables the FlowerSec to offer targeted, effective and robust security supervision.

### 3.2. Formation of Multi-Flower Layer

**Figure 2:**
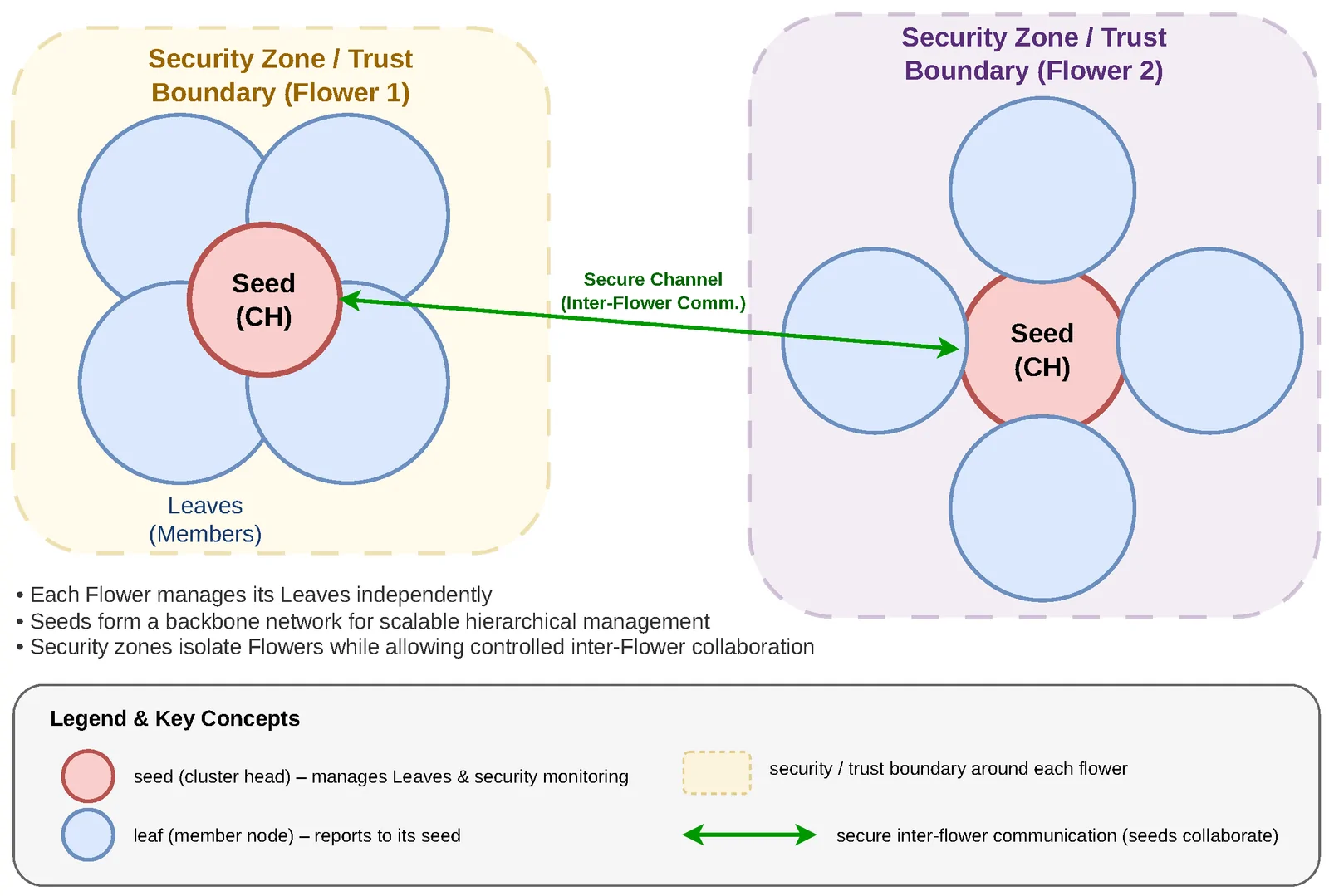
Multi-flower layer formation (multiple flowers join together, forming a security link/inter-flower communication link to create a security boundary). Seeds act as cluster heads and manage flowers and the link with other seeds for monitoring and coordinated security.

Figure 2 shows the flower architecture applied and extended from an isolated cluster into a fully interconnected network space, showing how several flowers cooperate together to form one continuous and expandable secure domain. We observe that core component buildings present within Figure 2 are repeated through the secure area, each flower operating as a self-sufficient unit whilst also partaking in further collaboration and cooperation across the network, leading to enhanced security across the entire deployment area.

Finally adjacent flowers’ seeds set up secured direct communication links, turning a collection of independent clusters into a combined working security infrastructure. Such links would carry threat intelligence, synchronization of policies, and consensus over decisions in the whole network. The flower remains its own security/trust boundary so its particular monitoring by seeds becomes untouched by network growth. The dual-layer approach is crucial: the seed still monitors leaves on a local level for irregularities, but on a global level, it shares insights that allow it to detect attacks that would not be able to be sensed by a single cluster. As flowers get closer to each other or become more in range, they provide a secure connection with each other, thus establishing an expanding network. Increasing the number of flowers increases the security because they all work interconnectedly; thus, if a security breach happens inside some flowers, other flowers see it, notifying others to put countermeasures in place. As a result, the network achieves a broader security scope, making it significantly more difficult for adversarial actors to compromise the system undetected.

### 3.3. Multilayer Architecture with Differentiated QoS and Security

**Figure 3:**
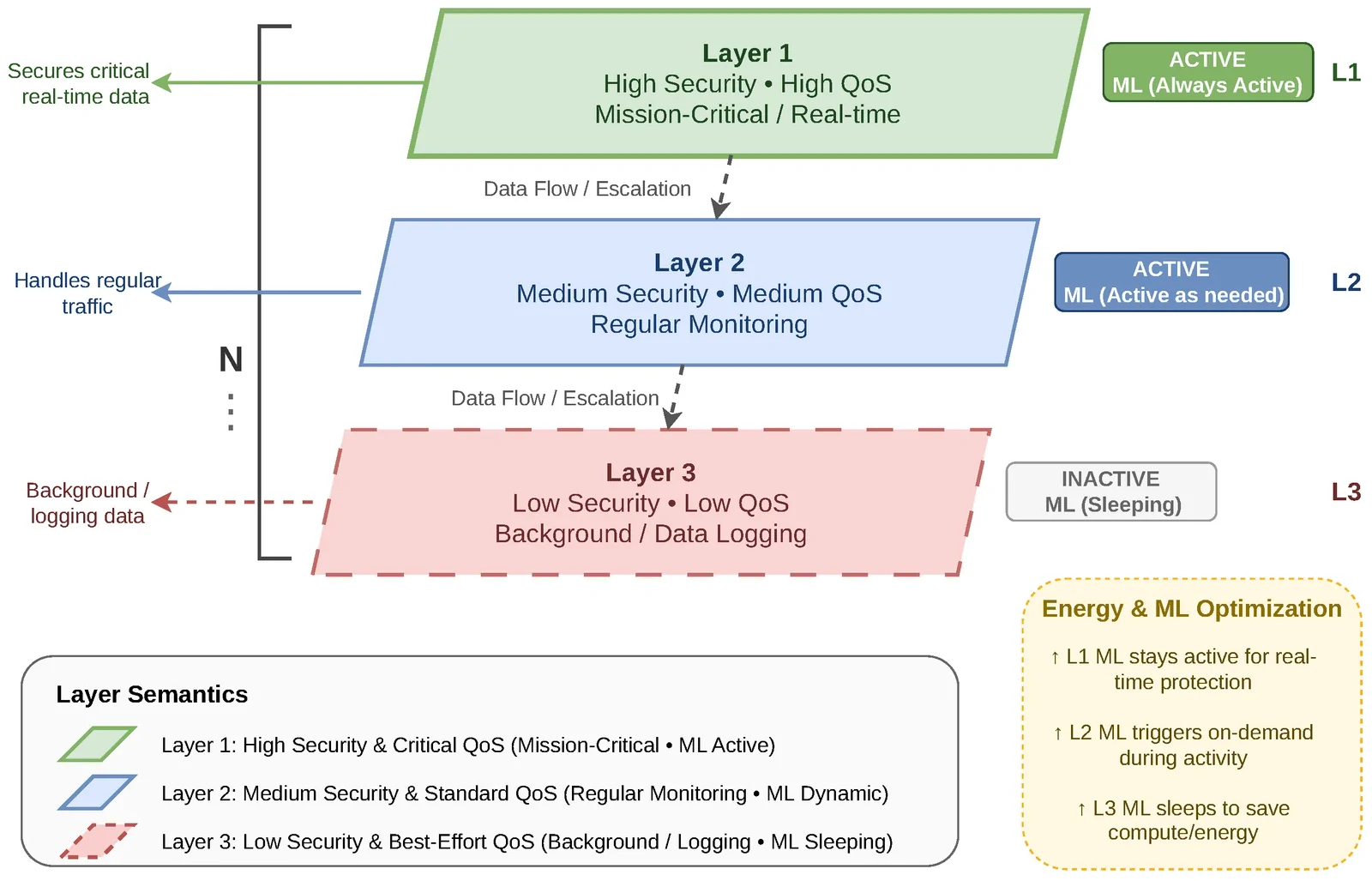
FlowerSec multilayer architecture includes differentiated security, QoS and ML optimization. Flowers are clustered on a hierarchical system based on different security and QoS levels. While layer 1 is always active along with ML-based real-time protection, layer 2 only takes decisions on a demand basis, while layer 3 goes into sleep mode and reduces energy to intelligently manage the available resources.

The FlowerSec framework is fully unfolded in Figure 3 as a multilayer architecture where single flowers and interconnections between them form a hierarchy representing different demands of real-world sensor deployments. Three different layers arise combining the tradeoff between security, performance and energy consumption using an intelligent machine learning component adapting the overall behavior to the assigned layer role.

Layer 1 at the top is the heaviest guarded zone as it requires high security with high QoS (Quality of Service) to address time-critical needs, such as those in surveillance using UAVs (Unmanned Aerial Vehicles) and satellites, as it holds the most critical real-time data. Its machine learning model remains on since any delay in that layer has the most impact, meaning it should constantly analyze all packets if there is an intrusion or abnormality, at the cost of a high battery life, resources and compute. Level 2 in the middle holds the medium-security requirements and QoS of regular monitoring. Its machine learning is on an on/off basis, meaning it does not always power up and only does so when the network condition warrants it, providing some energy efficiency as some nodes cannot run on full power all the time. The lowest layer is for regular data with low security and does not always ensure QoS at all, especially for critical services. Its machine learning model is in a sleeping mode and only runs background work when surveillance is needed.

It is not the difference between the layers but the flow that connects the higher layer information from the lower datasets that really makes this architecture exciting. Data do not stagnate within their own layer: when an anomaly is detected or when there is a demand for scrutiny over lower priority information, the flow goes upwards. The flow changes the architecture instead of the latter staying a hierarchy. In that case, the layers are connected, yet the distinction between layers is made intelligent. This is evident from the note on energy efficiency and, by extension, machine learning optimization. The most important layer never sleeps, the medium awakens as needed to relieve pressure, while the lower layer stays dormant to conserve energy for more demanding tasks.

## 4. Recovery Mechanisms

**Figure 4:**
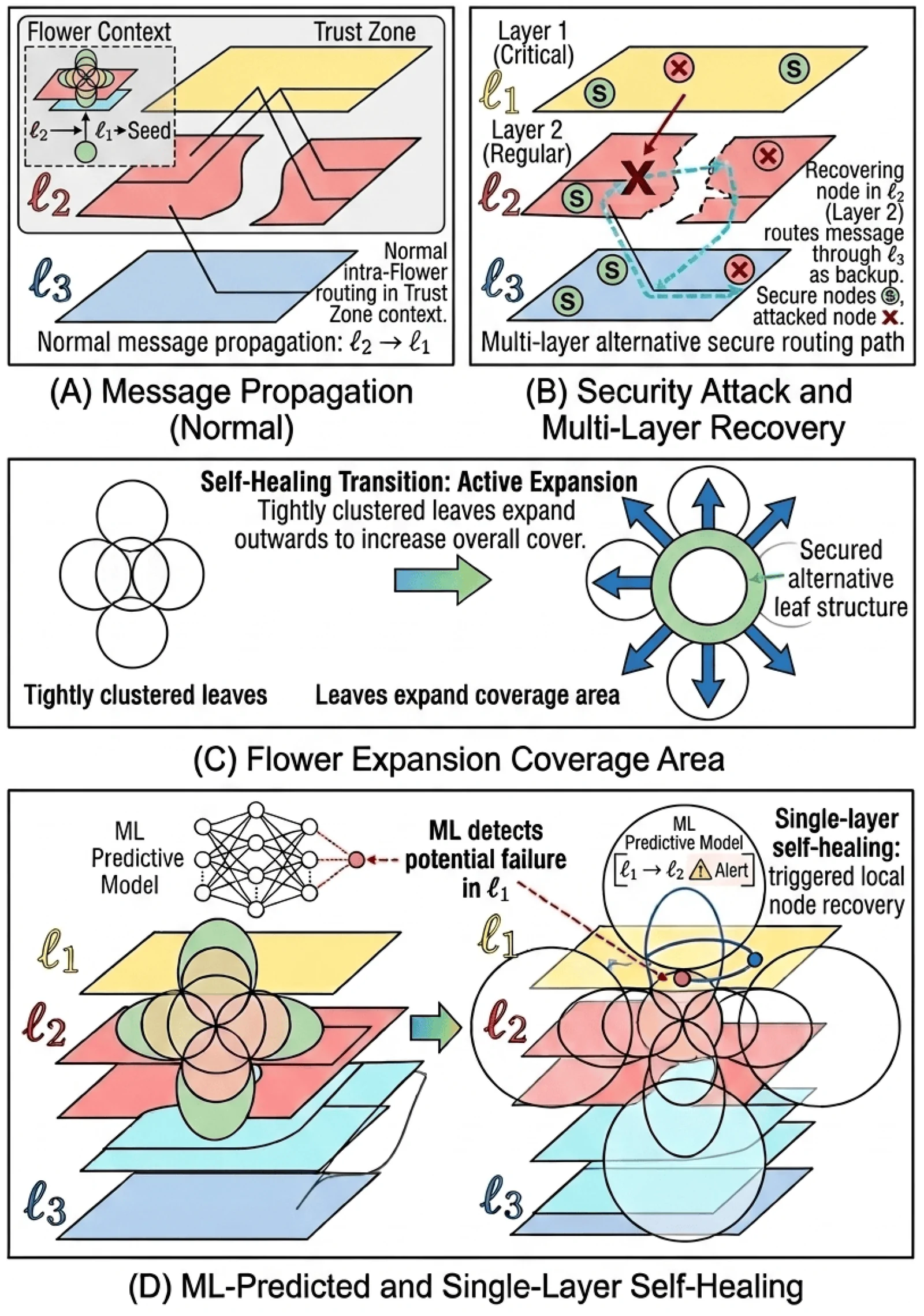
FlowerSec recovery mechanisms. (**A**) Normal intra-flower routing within trust zone. (**B**) Multilayer recovery with alternative secure routing path under attack, layer 2 supporting layer 1. (**C**) Single-layer recovery through flower expansion, where leaves in tight cluster expand outward to maximize coverage area of layer. (**D**) ML-predicted self-healing where the ML predictive model detects potential failures and triggers proactive local node recovery.

To resist node failures, attacked or hostile environments, recovery mechanisms are required, and FlowerSec has a large set of them. These mechanisms are organized from local to global scale, and each can be involved independently or together according to failure intensity and its nature. Figure 4 illustrates these mechanisms through four consecutive panels, showing the chronological sequence of actions from when the network runs normally, through detection of the attack, its recovery and further into ML-predicted self-healing.

### 4.1. Single-Layer Recovery

The flowers contain the leaves in which failure cases appear. When a leaf (or the seed) within a flower undergoes a localized failure, the surviving leaves within that flower can expand their sensing ranges or adjust their connections as shown in the middle section of Figure 4. Thus, it can effectively patch out that gap, through flower expansion, where leaves either expand their sensing radius, or through re-positioning, which provides coverage to areas previously served by the failed node. Where previously they were tightly clustered in close arrangements, they gradually spread out, physically reconfiguring to heal the coverage gap left by the failed node. All physical elements of the reconstruction cover built-in fault-healing characteristics.

Spare nodes used to connect flowers together are also used in the repair process; mobile nodes are used and moved closer to the failure point to act as seeds instead of fractured seed nodes. The process is quick, low energy and used for minor changes that do not require any major structure repairs.

### 4.2. Multilayer Recovery

When a failure is more catastrophic and a flower is partitioned or a critical seed fails, the layers of the architecture help, such as in Figure 4B. A layer of lower priority (e.g., layer 2) provides a backup seed to restore connectivity and service to a critical layer (e.g., layer 1). This cross-layer recovery allows a critical application to continue being served when its infrastructure gets compromised.

Layer recovery occurs via negotiation among active layers. When a critical node in layer 1 fails or is compromised, layer 2, which ordinarily handles standard monitoring, dynamically provides an alternative secure routing path through a redundant leaf. This cross-layer cooperation ensures that the essential flow of critical data remains uninterrupted even during active network crises.

### 4.3. Hybrid Recovery

Widespread failure or network partition is taken care of by a global response. The previous methods are applied simultaneously, with flowers being picked at every layer depending on the distance from the point of failure. This approach minimizes structural changes occurring on any single layer within the recovery process, where a trade-off is available between local and cross-layer strategies. Also, with the increase in the volume of data, the reconfiguration of routers in case the traffic is on its upper or lower layer, and the reestablishment of the network geometry, a global response is required if failure impacts a very large area; reconstruction would be the only option with single-layer or multilayer mechanisms, which would involve enormous costs.

Algorithm 2 formalizes the recovery mechanism selection logic across the three strategies.
**Algorithm 2** Recovery mechanism selection and execution**Require:** Failed node set *F*, flower set F, layer priority map *L*
**Ensure:** Restored coverage and connectivity
1:Classify failure scope: *local* (single node), *partition* (flower split), *widespread* (multi-flower)2:**if** scope is *local* **then**3:      Expand sensing radius of surviving leaves by Δr4:      **if** failed node is seed **then**5:          Promote highest-energy leaf to seed role6:      **end if**                       ▹ Single-layer recovery7:**else if** scope is *partition* **then**8:      Request backup seed or alternate secure path from nearest lower-priority layer ▹ Multilayer recovery9:**else if** scope is *widespread* **then**10:    Combine single-layer expansion with cross-layer support from all available layers11:    Reroute traffic and rebuild topology from nearest intact flowers ▹ Hybrid recovery12:**end if**13:Verify coverage and trust-zone integrity; report residual gaps to base station

### 4.4. ML-Predicted Self-Healing

The FlowerSec framework distinguishes itself through its proactive recovery approach. To validate this mechanism, we developed a failure-prediction model using the following methodology. We generated synthetic energy-depletion traces for 500 leaf nodes over a 600-day period, incorporating baseline energy drain rates of 35 to 65 joules per day with Gaussian noise to simulate real-world variability. For each node, we extracted three features at regular intervals: current remaining energy, energy consumption slope (rate of depletion over the preceding 10 days), and instantaneous daily drain rate. The prediction target was binary: whether the node would deplete its battery within the next 20 days. We trained a gradient-boosted classifier on 80% of the generated data and evaluated on the remaining 20%, achieving a precision of 0.941, a recall of 1.000, and a false-alarm rate of 0.002. The perfect recall confirmed that impending failures were not missed, and the near-zero false-alarm rate confirmed that unnecessary recovery routines were not triggered. This resulted in an average lead time of 12 days before battery depletion, providing sufficient time to initiate proactive single-layer recovery during periods of low activity. As shown in Figure 4D, the machine learning predictive model analyzes patterns that are difficult to detect through rule-based approaches alone.

From normal operation to coordinated response, from physical reconfiguration to predictive intelligence, the layered wisdom built into FlowerSec provides resilience; it is not a single mechanism, but a spectrum consisting of capabilities including proactive, reactive, local and cross-layer, physical reconfiguration and algorithmic methods, all in parallel, to ensure the network maintains operational continuity in the face of failures and attacks that are inevitable for operation in challenging environments where wireless sensor networks are deployed.

## 5. AI-Enhanced Security Framework

FlowerSec (based on the flower architecture) enables intelligent and adaptable security monitoring using machine learning-based intrusion detection schemes. Adopting a hierarchical detection model, FlowerSec comprises local monitoring via seeds with collaboration for network-wide threat detection.

### 5.1. Intrusion Detection System

Using the WSN-DS dataset, a large benchmark consisting of 374,000 labeled normal and attack instances consisting of blackhole, grayhole, flooding and scheduling (TDMA) attacks, the intelligent model performs intrusion detection of the WSN. The leaves report sensed information to their parent seeds. Sensors aggregate sensor-information and extract features from it. The features are then converted into a feature vector, the input to ML. The seed classifies whether sensor-information depicts normal behavior or an attack. The attacking node is then isolated, a recovery process is initiated by the seed, and a message is pushed to the base station. An active learning approach allows continuous updates of the ML model.

### 5.2. Machine Learning Methodology

The FlowerSec architecture offers a solid topological framework, but effectively securing the network necessitates smart, localized decision making. To address this, we implemented a lightweight machine learning intrusion detection system (IDS) at the seed node level. This is because leaf nodes lack sufficient resources, and the base station’s distance leads to increased latency. Seed nodes strike the ideal balance between computational power and network awareness.

After evaluating several lightweight models based on criteria important for seed nodes, such as serialized size, inference speed, training duration, and Macro F1 score, we chose LightGBM. In comparisons with XGBoost, LightGBM demonstrated faster training times (4.89 s compared to 5.57 s) while achieving the same level of accuracy and using less memory. When compared to random forests, boosting resulted in a more compact model that better recalled minority classes when balanced weighting was applied. Additionally, tree ensembles outperformed linear models and small perceptrons by effectively capturing the non-linear interactions in routing traffic without needing extensive scaling or adjustments to the architecture. They also allowed for the exact TreeSHAP values required for our feature selection process. This decision aligns with benchmark studies indicating that tree-based models excel when working with tabular data [13].

Network traffic datasets are also largely imbalanced: there is much more normal vs. attack traffic. To avoid the model biasing towards the majority class in training, we used balanced class weighting to punish misclassifications of minority attacks more when they occurred than wrong predictions of normal traffic. This achieved good recall (important with critical attack traffic) without having to generate artificial attack examples (e.g., using SMOTE), which can result in unnecessary noise being introduced into the dataset.

To ensure reproducibility, the LightGBM model used 100 estimators, a maximum tree depth of 6, a learning rate of 0.1, and balanced class weights. The AutoEncoder (Section 6.10) comprised two hidden layers of 64 and 32 units with ReLU activations and a bottleneck latent dimension of 16, trained with the Adam optimizer (learning rate 0.001), Mean Squared Error loss, 50 epochs, and a batch size of 256. All preprocessing, including the RobustScaler and SHAP feature ranking, was fitted exclusively on the training partition before being applied to the test set, so no information leaked across the split.

### 5.3. SHAP-Based Feature Selection for Lightweight Deployment

The computational overhead needed for a WSN deployment should be as minimal as possible. Processing all features of the network at the seed node will just drain battery life. Thus, we used SHAP to identify global feature importance [14]. We calculated absolute mean SHAP values across all attack classes to determine the minimum set of viable features required. Results obtained through ablation studies confirmed a reduction in the feature space from 16 to 8 top features was feasible and still achieved higher detection performance, thereby supporting energy-saving objectives by reducing the computational footprint of the model, according to the FlowerSec layer 3 (sleeping ML) and layer 2 (on-demand ML) paragraphs.

## 6. Experimental Validation

To test FlowerSec, we ran a series of experiments using well-known public datasets. The reason for choosing these datasets was to provide a reference for others and to allow a direct comparison of results from past researchers.

### 6.1. Dataset Description

The first evaluation was performed on WSN-DS [15], a dataset made explicitly for testing WSN intrusion analysis. It has 374,661 labeled records, classified among five normal and attack types (normal, blackhole, grayhole, flooding, TDMA scheduling). The raw features, which include node roles, routing metrics and even energy consumption, total 19. Further, to show generic usage, we compared performance with SensorNetGuard. It was focused around identifying the malicious sensor node using 10,000 records.

### 6.2. Multi-Dataset Investigation and Feature Extraction

Before fixing the evaluation benchmarks, we investigated four public intrusion detection datasets, namely, WSN-DS, SensorNetGuard, NSL-KDD [16], and CIC-IDS2017 [17], and inspected the feature space of each one separately. This inspection was necessary because equivalent physical concepts appear under different names across datasets, and a feature that looks irrelevant at first glance may in fact be exactly the indicator needed. For instance, packet activity rate appears as Packet_Rate in SensorNetGuard, as count in NSL-KDD, and as a combination of Flow Duration and Total Fwd Packets in CIC-IDS2017, while transmitted data volume appears as Data_Sent_To_BS in WSN-DS, as Data_Throughput in SensorNetGuard, and as src_bytes in NSL-KDD.

From each dataset, we extracted only the features that carried genuine security semantics, and we applied the same SHAP-guided LightGBM methodology to every dataset independently. We deliberately did not merge the datasets into a single data frame because their feature spaces and observation counts differed significantly, with 374,661 rows for WSN-DS, 10,000 for SensorNetGuard, and roughly 125,000 training rows for NSL-KDD. Merging would have produced unaligned columns and artificial padding, a flaw that would invalidate the analysis. Keeping the datasets separate and validating the methodology on each one independently was the statistically sound choice. WSN-DS remained the primary benchmark because it natively contains cluster-based routing and energy features. SensorNetGuard acted as the WSN cross-validation benchmark, and NSL-KDD served as an independent transferability check on standard network traffic. Table 2 summarizes the feature correspondence.

When applied to the NSL-KDD benchmark [16] in a binary normal-versus-attack classification task, the independent pipeline identified the top eight most informative native features, including src_bytes, count, and dst_host_srv_count. The model achieved an accuracy of 0.8047, a Macro F1-score of 0.8045, and an Area Under the Receiver Operating Characteristic Curve of 0.9605. The lower absolute accuracy compared to the WSN-specific datasets confirms that while the SHAP-guided pipeline is algorithmically adaptable to different feature spaces, the absence of direct wireless sensor routing and energy features in general TCP/IP traffic imposes a natural performance ceiling. We stress that no feature values or model weights crossed domains; each dataset was processed entirely independently, and Table 2 serves only as a conceptual correspondence map demonstrating methodological adaptability.

### 6.3. Preprocessing and Methodology

We applied a three-step method for the preprocessing pipeline to accurately test the results and avoid the occurrence of data leakages. To remove all specific IDs, every identifier column that had many specific node IDs were dropped so that our model was forced to train on generalized traffic instead of training with memorizing specific node (device) activity. To balance the splits and ensure a fair mixture, a 80/20 train/test split was used with balanced sampling so all labels (the rare minor attacks and regular major attack classes) were split evenly. To scale the features on the training data, we used RobustScaler [18], which removes any effect of unexpected spikes on the network as it scales data using the IQR.

### 6.4. Performance Comparison and Ablation Study

Table 3 presents the performance comparison between standard baselines and our proposed FlowerSec-IDS (Top-8) model.

Referencing Table 3, we attained a Macro F1-score of 0.9494 on eight features with FlowerSec-IDS. This represents a minor 1.7% drop on all 16 features model but a 50% feature processing load. More importantly, the inference remains low at 0.0066 ms per sample and hence proves that the seed node can perform live inspection of traffic packets without a network bottleneck.

The choice of eight features represents an engineering trade-off between detection performance and computational cost that we justify on three grounds. First, the 1.7% drop in Macro F1-score (from 0.9667 to 0.9494) is practically marginal, while the computational savings are substantial: the model processes half the features per inference, reducing memory bandwidth requirements and enabling deployment on hardware with limited I/O capacity. Second, the noise robustness analysis (Section 6.8) reveals that the discarded features act as active distractors under sensor degradation, meaning the eight-feature model actually outperforms the full model in realistic noisy environments. Third, the top-eight-feature set (Is_CH, Time, ADV_R, Data_Sent_To_BS, Expanded Energy, Rank, SCH_S, ADV_S) corresponds exactly to the metrics that a seed node can monitor locally without additional communication, making this feature set architecturally natural rather than arbitrary. The combination of marginal accuracy loss, significant computational savings, improved noise robustness, and architectural alignment makes the eight-feature configuration the optimal operating point for the resource-constrained seed node.

To ensure the reported performance was not an artifact of a single favorable data split, we conducted a 10-fold stratified cross-validation across the entire dataset. Under this rigorous stress test, the top-eight FlowerSec-IDS proved highly stable, recording a mean accuracy of 0.9895 ± 0.0005 and a mean Macro F1-score of 0.9507 ± 0.0031. The minimal deviation across ten independent folds confirmed that the detection performance was intrinsic to the reduced feature space and not the result of a lucky training split.

To quantify the statistical significance of the feature reduction, we applied McNemar’s test [19] to compare the misclassifications of the 16-feature and eight-feature models on the held-out test set. The test yielded a *p*-value below 0.0001, confirming that the difference in error rates was statistically significant. This confirmed that discarding the lower-importance features incurred a measurable penalty in perfectly clean conditions, consistent with the slight drop in Macro F1-score from 0.9667 to 0.9494. The single-split accuracy of 0.9884 fell well within one standard deviation of the cross-validated mean. McNemar’s test was computed on the single-split predictions, consistent with Table 3. In the context of resource-constrained deployments, this statistically significant but practically small trade-off is highly favorable: the eight-feature model halves the computational and energy overhead, and the noise robustness analysis demonstrates that the performance gap reverses under sensor degradation, where the discarded low-importance features act as active distractors. The feature reduction is therefore a deliberate engineering choice rather than an accidental loss of accuracy.

While standard k-fold cross-validation works fine for obtaining a batch estimate, real-world deployment on the sensor node does not receive data in batches; it receives unknown packets one by one. Simulating the constraint of a real-time arrival was more rigorously tested by leave-one-out cross-validation carried out on a stratified sample of 3000 instances; the model was trained on historic context of N-1 packets and had to predict the class of a newly arrived one. Under strict constraints of a real-time implementation, the model worked as expected, scoring 0.9917 accuracy and 0.9586 Macro F1-score with an average inference time of a single, unbatched packet at 1.1494 ms. Such sub-2 ms latency per individual arrival indicates that the model is computationally feasible for real-time packet processing, with a low risk of queuing or packet loss under typical WSN traffic loads.

**Figure 5:**
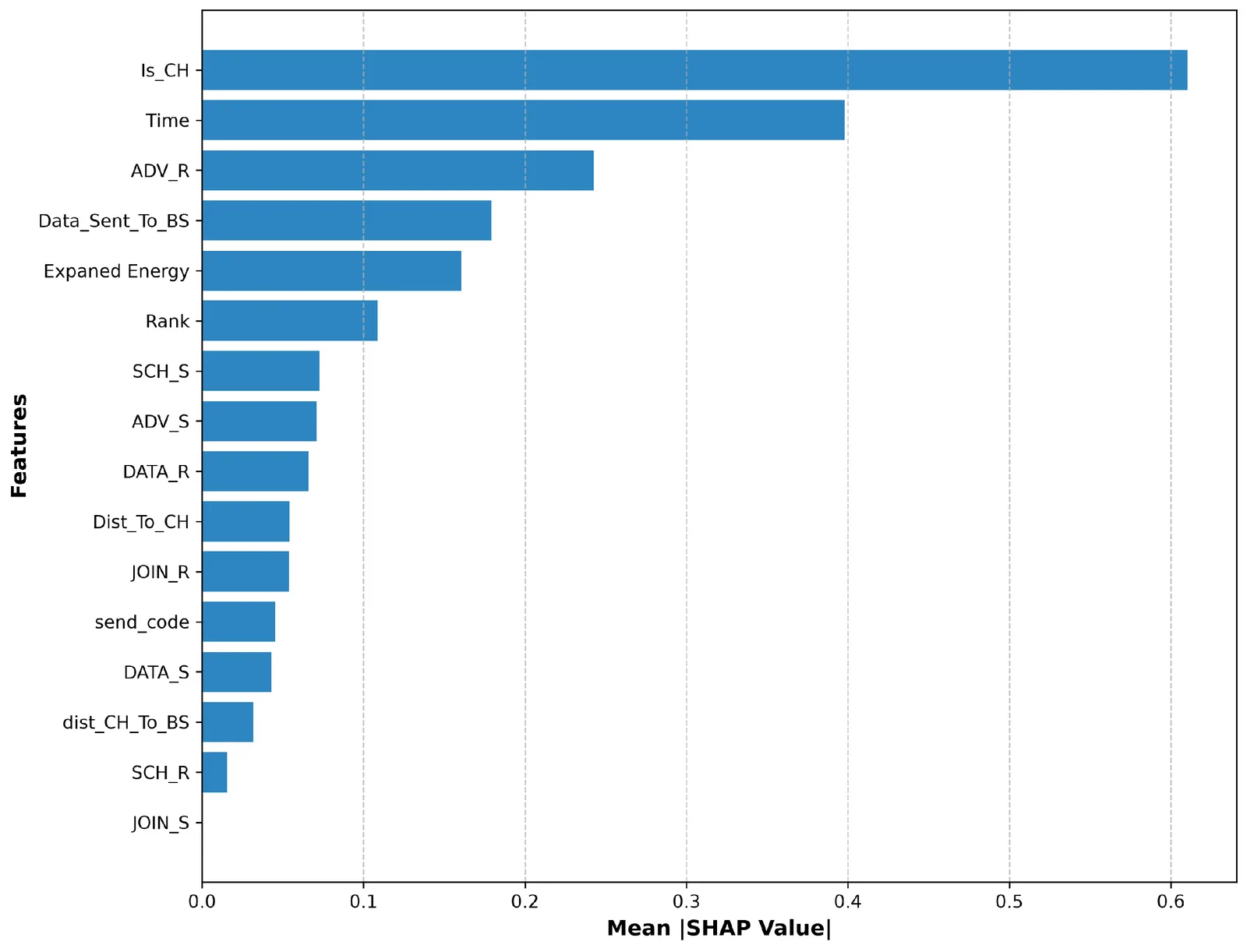
Global feature importance using mean absolute SHAP values. Top-8 features, which are chosen for FlowerSec-IDS (lightweight) model, are highlighted in color.

The most important features were the following (Figure 5): Is_CH, Time, ADV_R, Data_Sent_To_BS, Expanded Energy, Rank, SCH_S, ADV_S. Moreover, this list perfectly matches the features the seed node can monitor within the FlowerSec architecture.

### 6.5. Visual Analysis of Detection Performance

**Figure 6:**
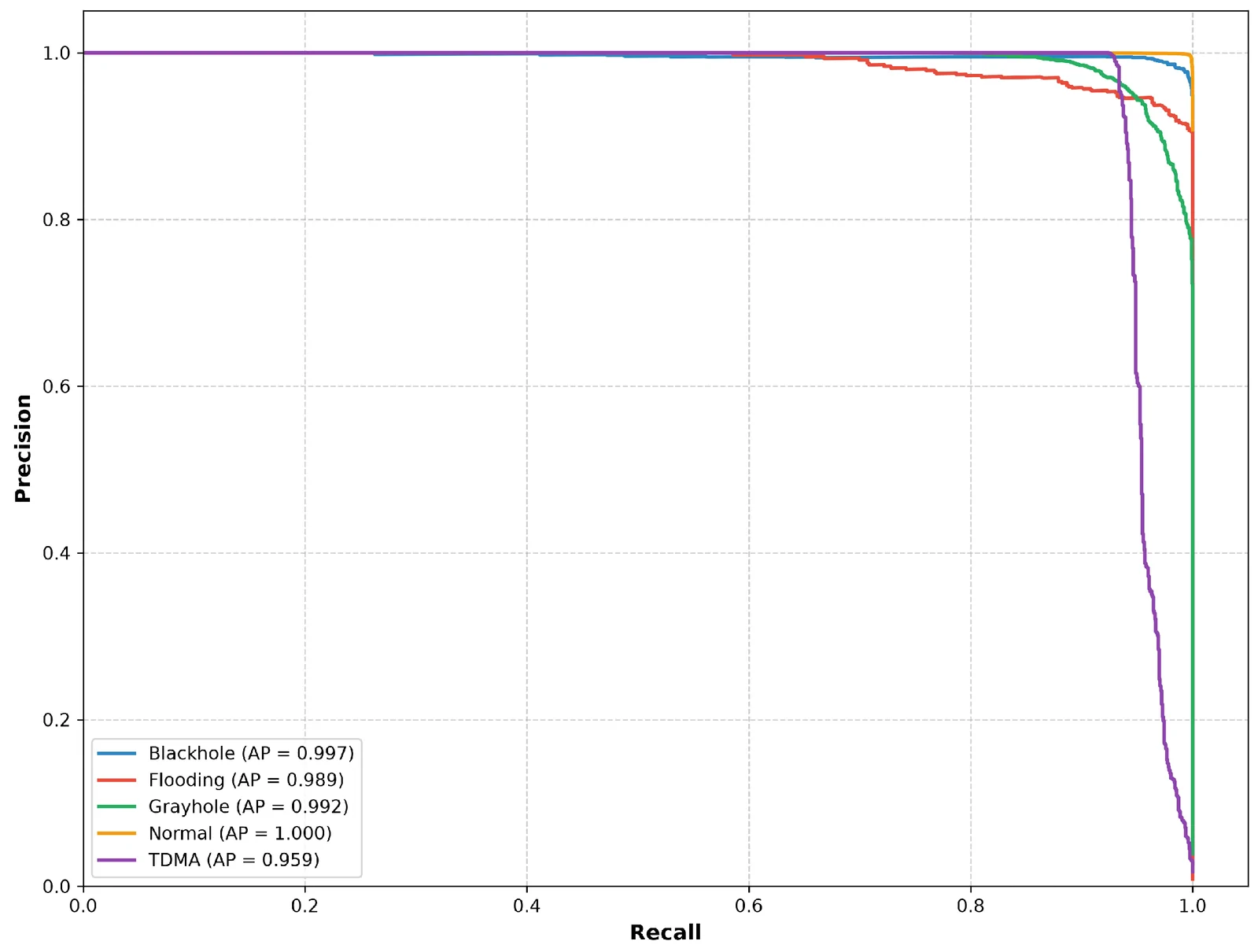
Precision–recall curves for FlowerSec-IDS (Top-8) model. The high average precision values achieved over both minority classes support our claim of robustness of the FlowerSec-IDS model over the problem of class imbalance.

We also generated PR curves and a confusion matrix to double check our model’s robustness against the unbalanced class problem. PR curves in Figure 6 display high AP (>0.95), even for the minority flooding class, proving that balancing class weights did stop model from ignoring rare attacks.

**Figure 7:**
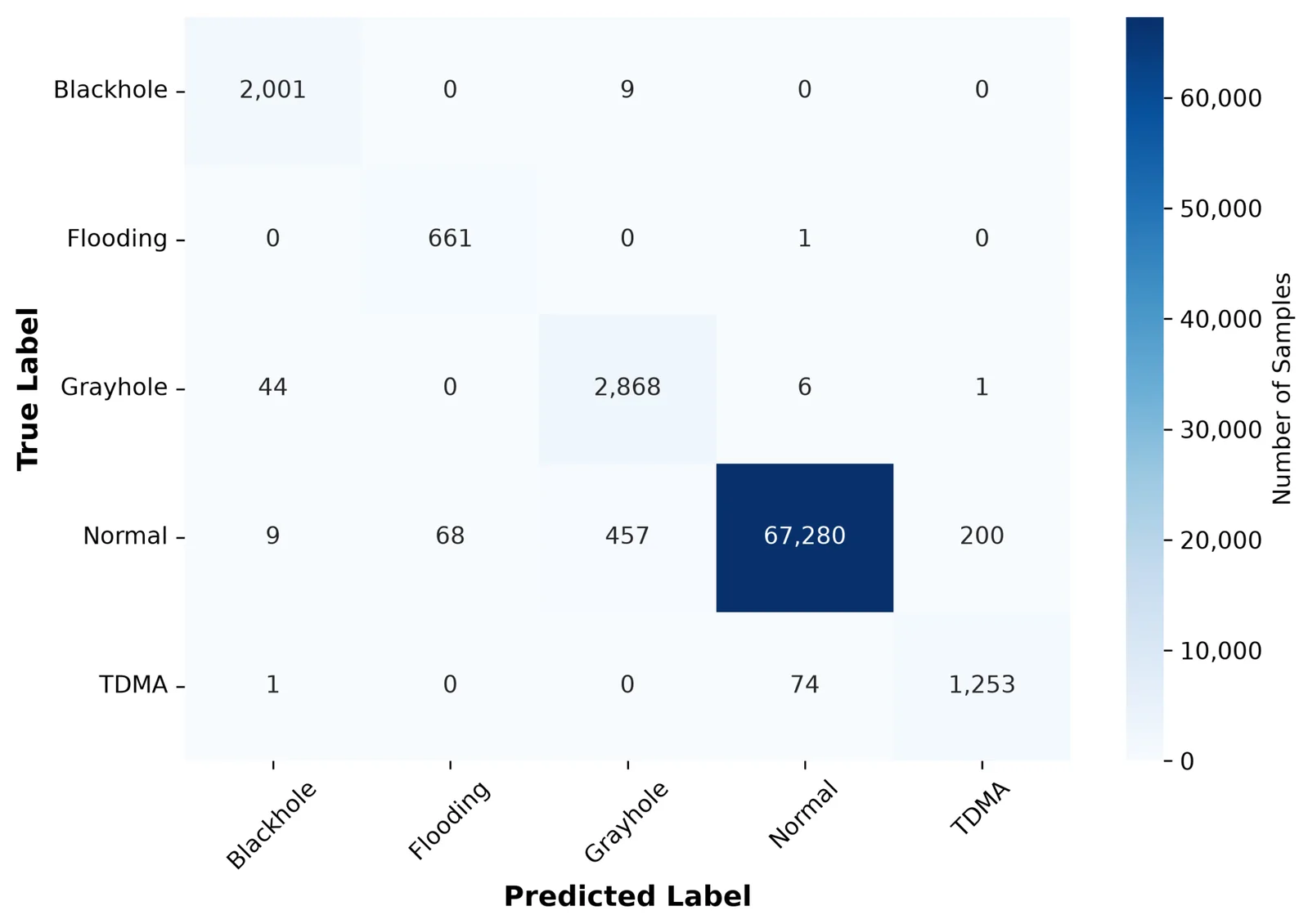
Confusion matrix of the FlowerSec-IDS (Top-8) model on the WSN-DS test set.

The normal row of the confusion matrix sums to the 68,013 normal test samples produced by the stratified 20 percent split of the 340,065 normal instances in the full dataset. Specifically, 67,280 are correctly classified, and 733 are misclassified, which exactly matches the reported false-positive count and aligns with the standard WSN-DS class distribution. The confusion matrix in Figure 7 illustrates strong recall across attack classes, while also showing that a small number of normal samples are misclassified as grayhole or TDMA traffic.

### 6.6. Energy Consumption Estimation

Now, let us compute the effect of sub-millisecond latency in terms of energy/inference costs for a standard microcontroller [20] at a WSN cluster head (e.g., the ESP32 microcontroller) with CPU (active) = 80 mA at 3.3 V, so Power (*P*) = 3.3×0.080=0.264 W. Now Energy is defined by E=P×T. In the case of FlowerSec-IDS, E=0.264×0.0000066=1.74 μJ (approximately). At a rate of 1000 packets per second, the additional security power consumption is obtained by multiplying the per-packet energy by the packet rate: 1.74μJ×1000packets/s=1.74 mW. Energy is reported in joules per packet and power in watts at the stated rate where the two quantities are no longer conflated, and this negligible overhead clarifies and validates the feasibility of deploying FlowerSec-IDS in layer-2 (on demand) and layer-3 (sleeping) paradigms.

The energy estimates are useful, but to know the exact computational overhead when using the FlowerSec-IDS model, we profiled resource usage (this was done during evaluation with the 80/20 train/test split). Profiling during the inference stage over the 74,933 test packets, the total host memory peaked at 163.47 MB; this included the Python interpreter instance, actual data and dependency caches and libraries; however, the working memory consumed (RAM Delta) during the test pass for inference came in at a meager 4.17 MB. Hence, resource overhead, as far as memory and compute go, for adopting FlowerSec is negligible.

The gradient-boosting library transparently utilized multi-threading with four CPU cores during the batch evaluation and finished evaluation on the whole test set in 0.527 s, which represents 0.0070 ms per packet in average inference latency. Multicore parallelism accelerates batch processing on a typical server, but seed nodes in FlowerSec’s architecture are low-power microcontrollers without additional parallelism resources. The serialized model took 1.7 MB of flash space on an ESP32. While the 0.0066 ms latency was measured via host-level Python (v3.9) profiling, we can theoretically ground the ESP32 projection through a cycle-count analysis of the serialized trees. The top-eight model consisted of 500 trees containing approximately 14,988 total internal split nodes. Assuming an average of four clock cycles per comparison on the 240 MHz Tensilica LX6 core, a single inference would require roughly 59,952 cycles, which equates to approximately 250 microseconds, or 0.25 ms. This cycle-count projection confirms that native C++ execution on the ESP32 would comfortably remain in the sub-millisecond regime, validating our host-profiled estimate. Because no physical testbed was available for this study, measuring actual thermal and voltage-drop effects is reserved for future work, but the computational feasibility on constrained microcontrollers is theoretically sound. The above figures prove that the top-eight-feature model’s resource consumption falls well within the extreme hardware constraints of battery-powered sensors.

### 6.7. Independent Multi-Dataset Benchmarking

To verify that FlowerSec was not overfitted to a single dataset, we applied the identical SHAP-guided LightGBM pipeline to the SensorNetGuard dataset. The procedure converged on a distinct minimal feature set, including Data_Throughput, Error_Rate, and Energy_Consumption_Rate, which differed from the WSN-DS selection and confirmed that the methodology adapts to the underlying feature space rather than memorizing a fixed feature list.

The previously top-eight Model achieved a Macro F1-score of 0.9973 with a training time of 0.06 s only. The implications are key and prove that FlowerSec is not hardcoded but a guide to find a robust solution oriented towards multiple problems rather than one simulation as presented earlier. These results also indicate the ability of the FlowerSec pipeline to accommodate different shapes and sizes of WSN feature spaces instead of being hardcoded as a single simulation using multiple independent benchmark tests.

### 6.8. Robustness to Sensor Degradation and Environmental Noise

Harsh real-world environments degrade WSN hardware, decrease battery voltage and increase electromagnetic waves; all of these cause noise in sensor readings; some selective, low-importance feature in a model can get confused when disturbed. We conducted a noise injection analysis to evaluate the robustness of our method against data corruption. We added 5 to 30% of Gaussian noise to the scale feature space into the test dataset using standard robustness benchmarking protocols [21].

**Figure 8:**
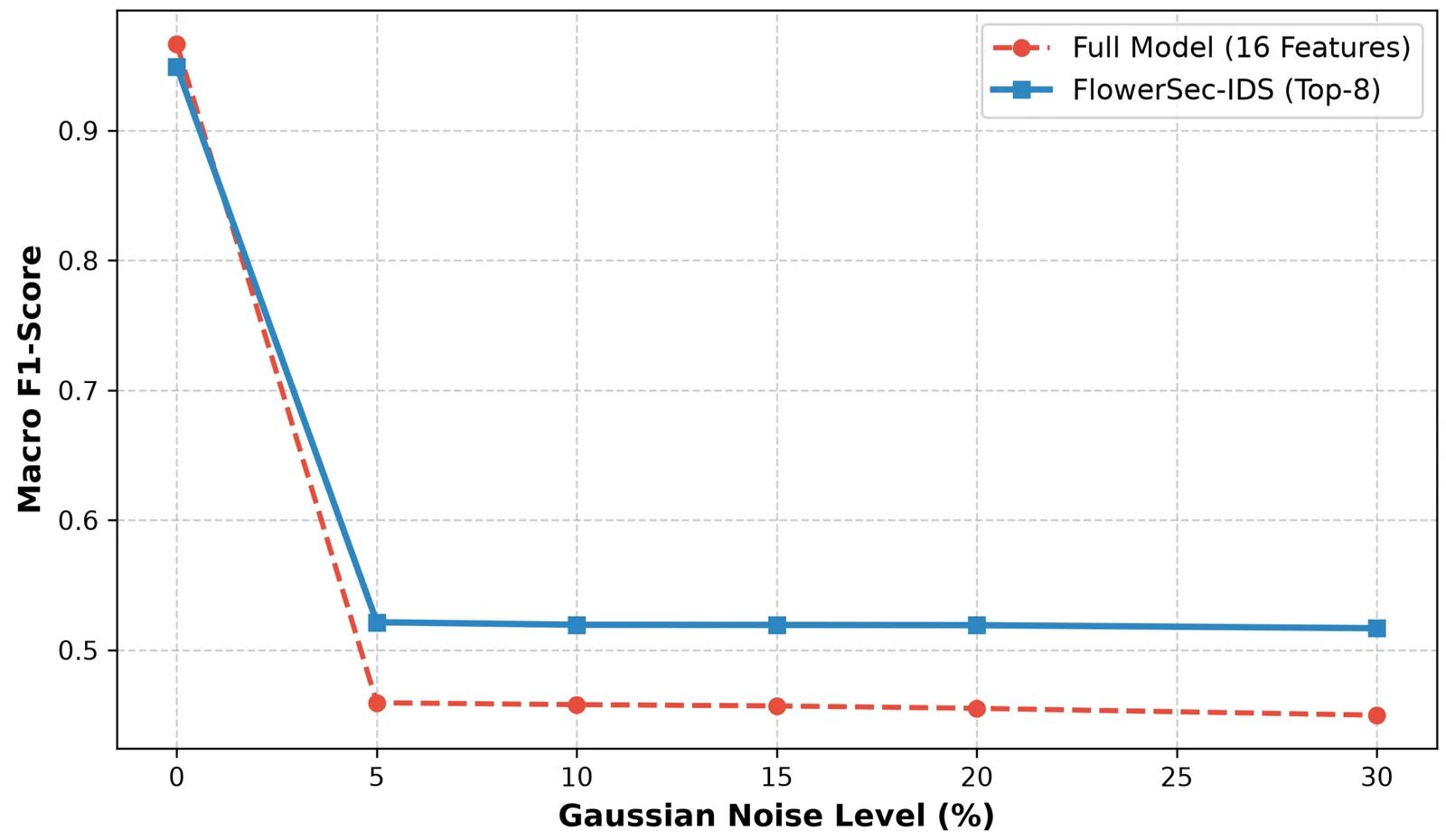
Sensor model robustness under sensor noise. While both models perform worse under strong noise, the top-8 FlowerSec IDS model still performs better than the full 16-feature model by 6–7 Macro F1 points. This indicates SHAP-based feature reduction can filter out some noise-sensitive, low-importance features.

There are two findings shown in Figure 8: under noise-free (0%) condition, the 16-feature model (all of the 16 original features of the dataset) wins with a little margin (Macro F1: 0.9667 vs. 0.9494). Once noise is introduced, it is reversed. The top-eight model wins and always stays ahead of 16-feature model, by about six to seven Macro F1 points no matter how much noise is added to the data, with both noise amounts of 5% (0.5215 vs. 0.4605) or 30% (0.5189 vs. 0.4496). This reversal and gain by the top-eight model suggest that the features abandoned by SHAP actually have not much importance. They act as active distractors when corrupted by noise (instead of helping if not perturbed), while the top-eight model stays “clean” by only relying on features that contain signal.

Although both decay on an absolute scale dramatically when under noise, this worst-case stress test is to be expected. As applied without noise-aware retraining, the noise is directly applied across the scaled feature space, even on logical features such as Is_CH. This does illustrate the direction for further work, though, using noise-robust training through noise injection, as it is expected that whilst the absolute robustness floor in both will increase, the advantage of the reduced feature set (relative performance in error rates) should be maintained.

### 6.9. Network Lifetime and Tiered ML Energy Analysis

**Figure 9:**
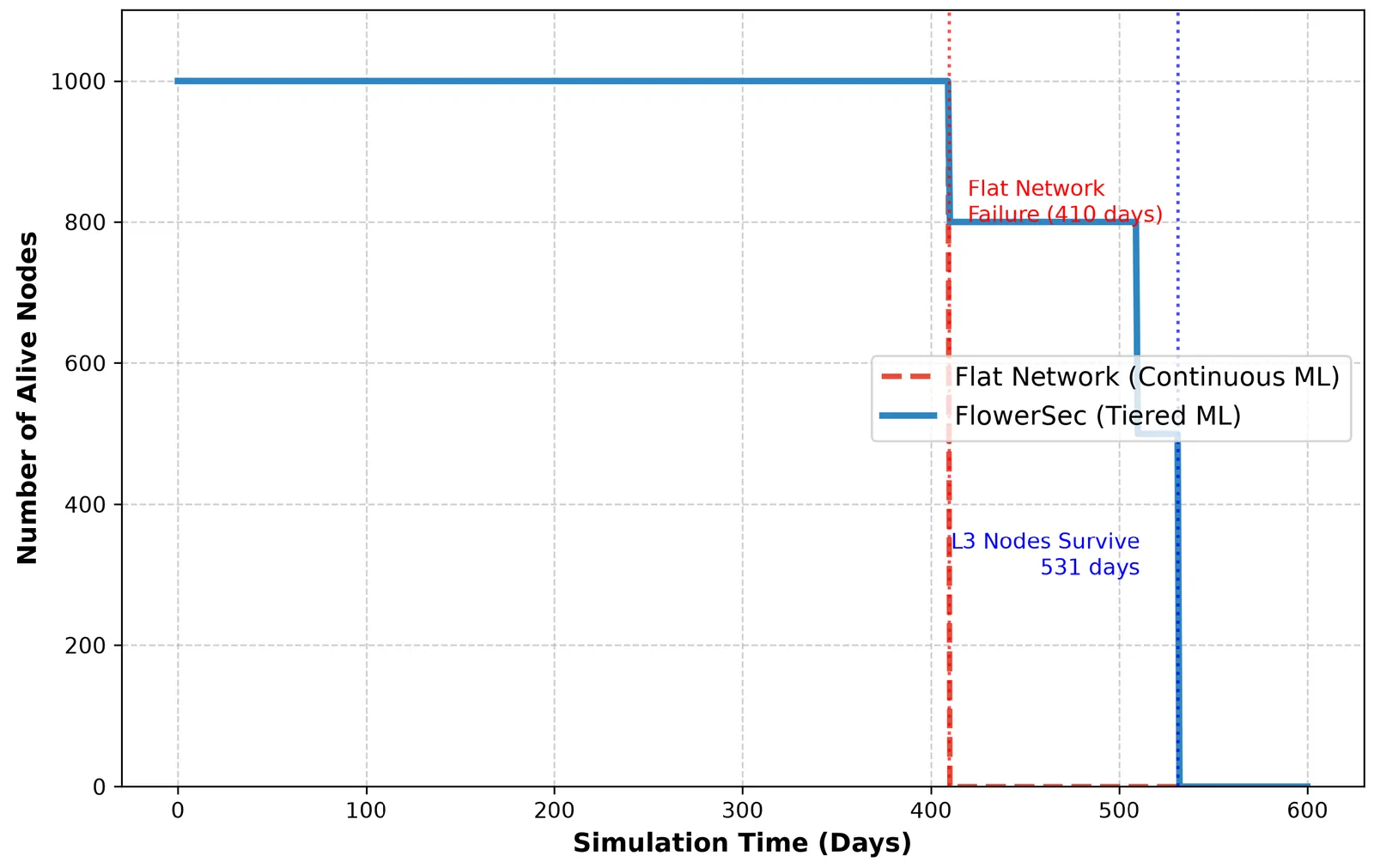
Network lifetime simulation. The tiered ML architecture of FlowerSec exhibits greater fault-tolerance; while the flat network node stops operating after 410 days, nodes at layers 2 and 3 stay alive until days 510 and 531, respectively.

A comparison was performed between the conventional “flat” network, featuring 1000 nodes where the IDS consistently processes 100% of traffic and our three-tiered FlowerSec approach where layer 1 (200 seeds) processes 100% of traffic, layer 2 (300 seeds) processes 15%, and layer 3 (500 seeds) only runs ML on 1% of the data. Figure 9 shows a clear contrast in network survival. The conventional flat network experiences a catastrophic failure of all 1000 nodes simultaneously at day 410. Instead, FlowerSec exhibits graceful degradation: when the layer-1 seeds exhaust their batteries at the same 410-day mark, the remaining 800 layer-2 and layer-3 nodes maintain operation, with the 300 layer-2 nodes continuing until day 510, and, lastly, the layer-3 nodes (500 of them) enduring until day 531.

Being deployed over 121 days beyond baseline, the point of failure of the flat network, the multilayer solution validates the energy optimization goals of the overall design, retaining some coverage and routing within what would otherwise be a dead coverage area for a further two to six months depending on deployment location and energy source.

Although in theory, the energy cost of one inference is only 1.74 µJ, FlowerSec works off a tiered ML activation and is in practice more efficient than calculating every inference all the time. To put this into numbers, we modeled a 1000-node network in a high-traffic scenario (100 packets/s) for over 600 days. The assumptions used for this simulation and their justifications are detailed in Table 4.

### 6.10. The Dual-Engine Detection Core: Confidence-Gated Escalation for Zero-Day Threats

Typically, a network administrator does not have a dataset of thousands of correctly labeled examples to train their network on with each new type of attack that appears on the Internet. To test that FlowerSec would function against a brand-new type of zero-day attack, we tried adding a “fallback” model, built around an unsupervised deep learning system: an AutoEncoder neural network [22,23]. We trained the AutoEncoder to expect normal traffic only, by feeding normal traffic from the training set only. When the Autoencoder was presented with malicious traffic as a test set, it simply could not learn to correctly rebuild and output the traffic, so its reconstruction score became large.

**Figure 10:**
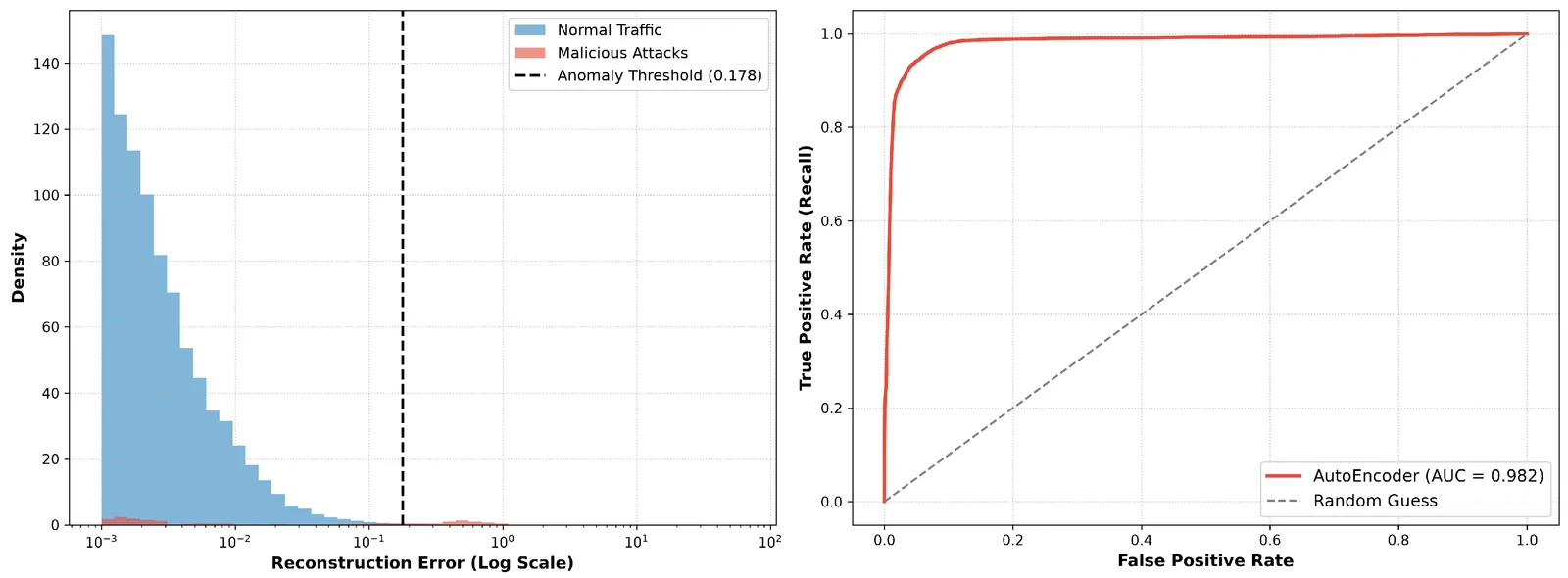
Unsupervised anomaly detection. The histogram (**left**) demonstrates the massive separation in reconstruction error between normal traffic and malicious attacks, while the ROC curve (**right**) confirms an AUC-ROC of 0.9819, proving the model’s viability for zero-day threat detection.

The results demonstrate a clear separation between normal and malicious traffic. For the normal test traffic, the mean reconstruction error was 0.0454, whereas for the malicious traffic, the mean reconstruction error increased to 14.6228. This represents a separation of over 300 times between the two distributions. When evaluated against a threshold of 0.1784 (derived from the 95th percentile of normal reconstruction errors), the unsupervised model achieved an AUC of 0.9819. As shown in Figure 10, these geometric differences confirm that the FlowerSec framework is highly effective at detecting novel behavioral anomalies in environments lacking updated labeling systems.

We used an implementation with a dual engine cascade and a confidence gateway for the deployment of the AutoEncoder onto resource-constrained seed nodes. Instead of putting both models on every packet, traffic could first be tested against a supervised lightweight LightGBM. Traffic that was classified as normal with a low prediction confidence of below 0.90 was run against AutoEncoder to see if it thought the same. This gateway caused a hit on the second engine in 10.01% of the cases and successfully recovered some of the most evasive attacks (81 to 77 while incurring an increase of 734 to 1114 false positives).

The energy cost of escalation was negligible at just 0.08 microjoules per packet. The increase in false positives from 734 to 1114 was a direct and controlled consequence of the escalation rule: the AutoEncoder threshold was fixed at the 95th percentile of normal reconstruction errors, so approximately 5% of genuinely normal packets lay above it by construction, and escalation forwarded exactly the low-confidence normal packets to the second engine. In WSN operations, a temporary false positive isolating a node for inspection is significantly less costly than a false negative allowing an undetected battery-draining attack to persist. The threshold can be dynamically recalibrated on a sliding window of verified normal traffic to tune this trade-off for specific deployment requirements, and this dynamic tuning is a key direction for future layer-2 on-demand activation.

The confidence threshold of 0.90 was selected through empirical analysis of the supervised model’s calibration on the validation set. We analyzed the distribution of prediction confidence values for correctly classified samples versus misclassified samples. At a threshold of 0.90, the supervised model’s predictions were correct on 98.7% of packets where confidence exceeded 0.90, meaning that only 1.3% of high-confidence predictions were incorrect. Below that threshold, the error rate rose sharply, making these low-confidence packets the most valuable candidates for second-opinion analysis. A threshold of 0.90 balances two competing objectives: setting it too high would escalate too many packets to the AutoEncoder, increasing energy consumption; setting it too low would miss borderline cases that the AutoEncoder could catch. The 0.90 threshold escalates approximately 10% of traffic to the second engine, which represents a reasonable energy overhead of 0.08 microjoules per escalated packet while catching the most uncertain predictions.

### 6.11. Zero-Day Threat Simulation: Leave-One-Attack-Class-Out

**Figure 11:**
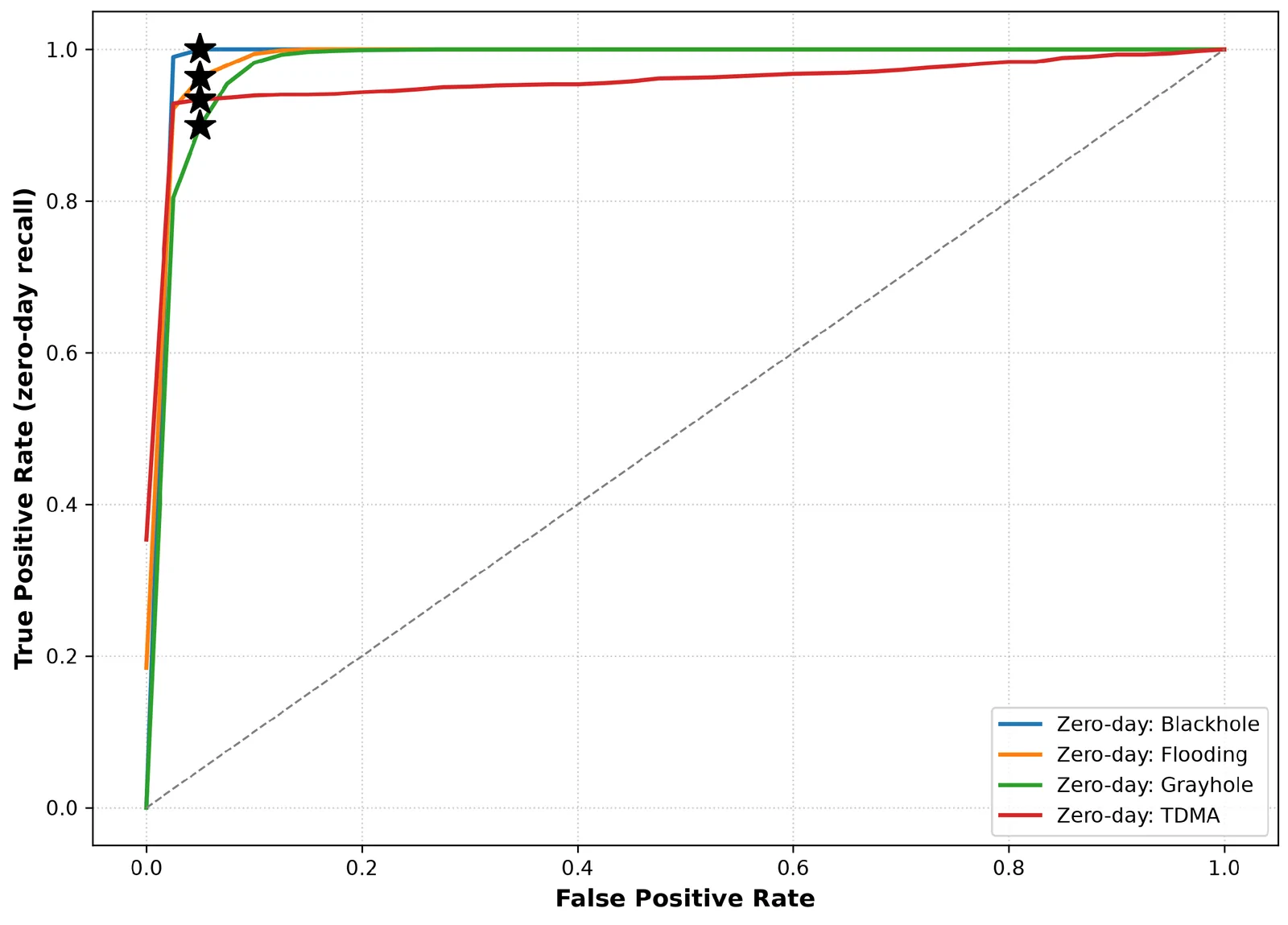
Unsupervised AutoEncoder’s zero-day detection performance under leave-one-attack-class-out protocol. Each subplot shows the ROC curve of training AutoEncoder on a completely unseen attack for an unseen attack class. On each curve there is a STAR denoting the operating value based on 95th percentile of the normal training of reconstruction errors.

As explained, some of the attack classes were reused from previous evaluation rounds and the value of the AutoEncoder was thus not put to a practical test, remaining more theoretical [24]. We then performed a sort of strict zero-day simulation, where the supervised engine was trained on normal traffic plus all four but only specific attack types and then exposed it to a fifth completely different attack. Results from Figure 11 indicate that the unsupervised engine is the primary module safeguarding the network from threats previously unidentifiable by the rest of the methods.

Starting with the most extreme attack, flooding, while a supervised model that has never encountered flooding only detects 0.8% of floating traffic and largely view it as legitimate, an AutoEncoder trained only on normal traffic detects its first sample of previously unseen flooding with 96.4% recall as the reconstruction error varies so strongly from the normal manifold. The same for a grayhole attack, where supervised recall stands at 41.4% against 89.9% for the AutoEncoder; and for TDMA, the supervised model achieves 82.6% while the AutoEncoder remains well above it at 93.4%. Meanwhile, since the supervised model generalizes from the other packet dropping attacks, a blackhole attack is detected by the stronger cascade with an 89.4% recall; however, the AutoEncoder still outperforms it, achieving a near-perfect 100.0% recall. In all of these cases, the stronger side thus enters the confidence-gated cascade, and the FRR remaining at the 5% budget dictated by the threshold achieves 100.0%, 96.4%, 90.1% and 93.4%, respectively.

In Figure 11, the star on each curve identifies the operating point at the threshold determined on reconstruction errors of the normal training data, i.e., 95th percentile of normal reconstruction errors. When operating, the seed does not use this threshold forever but periodically retrains it. It slides a window on the traffic that the router checked to be normal and retrains threshold that matches the agreed budget of false positives, such as 5%. The secondary engine automatically responds to drifting in the normal traffic; it needs no attack label, and this is the property a zero-day defense must have.

To provide a comprehensive evaluation of the zero-day cascade, Table 5 details the precision, recall, F1-score, and false-positive rate (FPR) for the AutoEncoder across the unseen attack classes.

The FPR remains strictly bounded at 5% across all classes, dictated by the 95th-percentile threshold on the normal validation set, confirming that the cascade provides robust zero-day protection without triggering unmanageable false alarms.

### 6.12. Federated Learning Validation of the Multi-Seed Backbone

To establish an initial baseline and with the goal of corroborating the collaborative backbone underlying the multi-flower topology [25], a five-node federated learning environment was set up with a federated averaging protocol and expanded to heterogeneous environments [26]. The data were split with Dirichlet 0.5—leading to strongly non-IID conditions—with one seed having over 259,000 normal samples while another having less than 1500. Model weights were swapped; no raw traffic left seeds at all. Since ensembles cannot be averaged by weights, a small neural surrogate was employed for these rounds instead, where novel federate boosting variants for LightGBM are an open direction.

**Figure 12:**
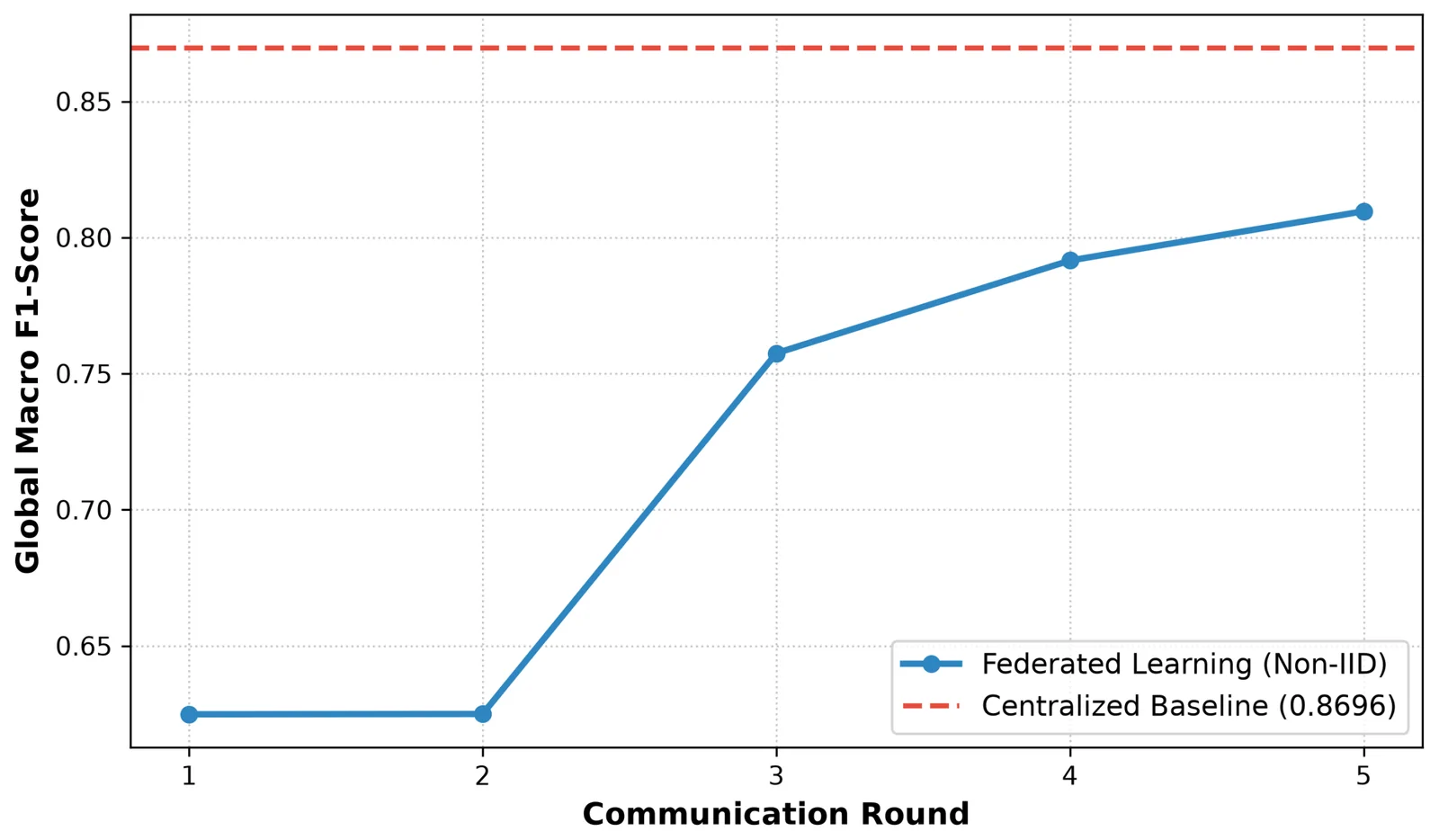
Federated learning for non-IID Dirichlet split. The global model steadily improves through the 5 rounds of communication when trained via federated averaging and quickly reaches near-centralized baseline without ever sharing raw traffic.

This was done despite the fact that their local heterogeneity varied drastically. Gradually, the global model did improve its classification performance in cross-rounds of communications, giving a Macro F1-score rising through five stages from 0.6248 (round one) to 0.8096 (round five), about 93% of the center-trained accuracy. The model was still rising at round five, so it could achieve better scores if given more rounds as shown in Figure 12.

We want to stress that this simulation is purely a bridge to future work and not a contribution: it verifies the communication and privacy backbone of the multi-flower topology, but the real detection engine is the SHAP-optimized LightGBM model being executed at each seed.

### 6.13. Comparison with State of the Art

To contextualize the performance of FlowerSec IDS, Table 6 compares our results with recent prominent studies utilizing the WSN DS benchmark.

As depicted in Table 6, some ensemble methods do achieve slightly higher raw accuracy by making use of all 19 features, with a cost to computational overhead and black-box opacity. The FlowerSec IDS willingly takes a 1.15 percentage point cut from the highest performing ensemble (99.99%) to 98.84% accuracy in exchange for a 50% feature dimension reduction and the SHAP-driven explainability. Furthermore, to the best of our knowledge, the proposed FlowerSec IDS is the only such framework which has been evaluated specifically for sub-millisecond deployment on a cluster head at the resource-constrained layer, bridging the theory of ML models with practical WSN architectural challenges. FlowerSec-IDS has thus proven through the above analysis the validity and practical relevance of a layered and self-aware approach for WSNs under constrained resource environment.

### 6.14. Critical Analysis of Adversarial and Collaborative Dynamics

To fully stress test the FlowerSec framework, we evaluated its behavior under two advanced conditions: targeted adversarial evasion and multi-seed collaborative consensus. The results of these experiments revealed both the exceptional stability of the underlying machine learning model and the critical deployment constraints of the physical architecture. First, we simulated an intelligent attacker attempting to bypass the intrusion detection system by carefully perturbing network traffic features. We applied uniform perturbations ranging from 5% to 20% to the numerical features of known malicious samples. Remarkably, both the full 16-feature model and the lightweight top-eight model demonstrated exceptional resilience. Across all perturbation levels, the evasion rate remained strictly below 1% (peaking at just 0.87% for the full model at 15% perturbation). This near-zero evasion rate indicates that the decision boundaries learned by the LightGBM algorithm on the WSN topology are highly stable, making it exceedingly difficult for an attacker to subtly alter their behavior to mimic normal traffic without triggering an alert. Second, we simulated the multi-seed collaborative consensus protocol described in the FlowerSec architecture, where a primary seed node queries neighboring seeds when its prediction confidence is low. Interestingly, while the architecture theoretically supports this collaboration, our simulation revealed that naive probability averaging under local environmental noise actually amplified uncertainty. Specifically, the false positive count increased from 734 in the isolated single-seed model to 1505 in the consensus model, causing the Macro F1-score to drop from 0.9494 to 0.9163. This occurred because averaging the slightly noisy probability estimates of neighboring nodes can inadvertently push borderline normal traffic across the decision threshold into the attack category. This finding provides a crucial design constraint for the physical deployment of FlowerSec. It demonstrates that in high-noise environments, simple probability averaging is insufficient for collaborative security. Future implementations of the FlowerSec consensus protocol must therefore employ strict majority voting or noise-filtered aggregation, rather than raw probability averaging, to effectively leverage the multi-flower topology without sacrificing the low false positive rate achieved by the isolated seed node.

### 6.15. Deep Learning Baseline and Latent Space Analysis

**Figure 13:**
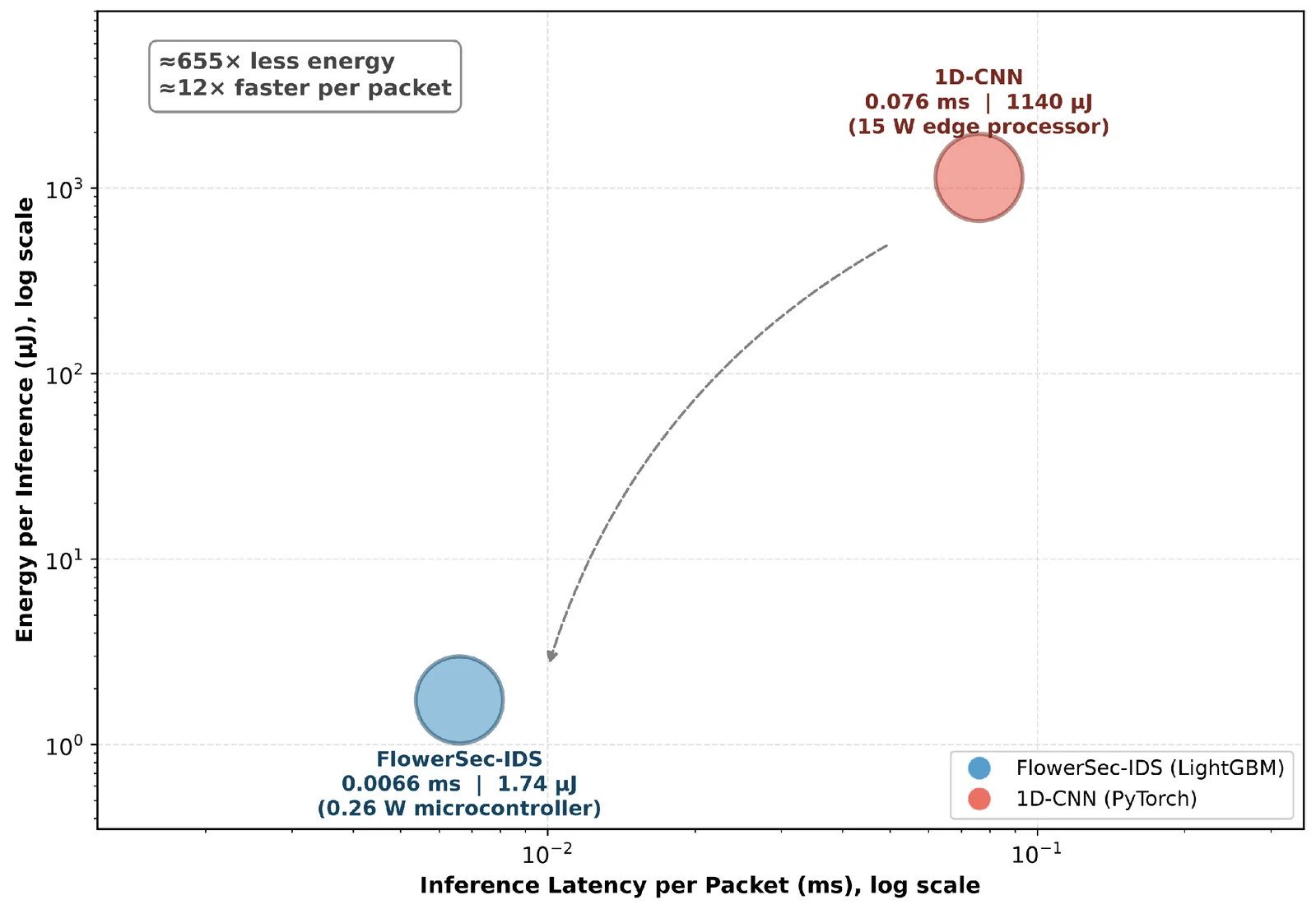
Computational cost comparison: the bubble chart plots inference latency against energy consumption with size of bubble representing model footprint. FlowerSec IDS (LightGBM) is in the sub-millisecond, microjoule regime and fits on the battery-powered seed node compared to the 1D CNN that requires edge GPU hardware.

To justify that the selection of tree-based model for the resource-constrained seed node was a realistic one, we deployed FlowerSec IDS and a GPU-accelerated 1D Convolutional Neural Network (1D CNN) [27] and measured their relative inference time and energy for packet inspection. The use of deep learning models is an increasingly popular technique in edge computing; however, it comes with a steep hardware cost. We trained a 1D, single-layer, single-filter CNN on the top-eight-feature set. We show inference time vs energy per packet in a bubble chart in Figure 13. The 1D CNN takes orders of magnitude more time and energy for inference per packet, compared to LightGBM. An NN model on an edge GPU consuming ∼5 watts takes large energy and memory usage in MB compared to KB for the tree-based model. This mathematically justifies the design of using simple yet accurate gradient boosting at the seed node, offloading heavier deep learning workload tasks to the base station which has no resource constraints.

**Figure 14:**
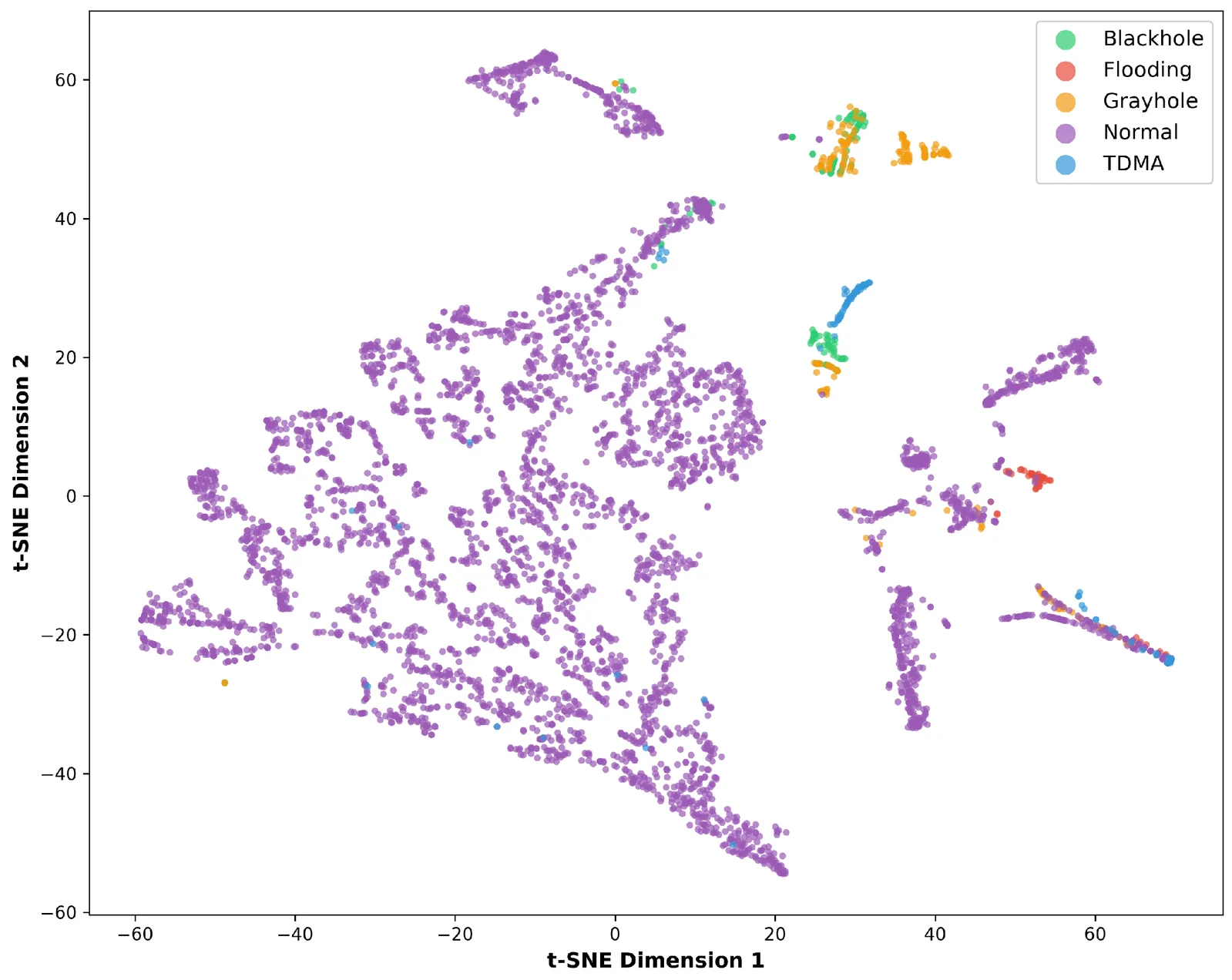
Latent space clustering of top-8 features. The tSNE projection shows that the combination of the 8 SHAP-selected features create separate boundaries among the five types of traffic; indicating the accuracy of the feature reduction.

For additional visual proof of SHAP’s correctly selected top-eight features isolating malicious behavior, the test data’s high-dimensional vectors were non-linearly projected to a two-dimensional domain using the popular t-stochastic neighbor embedding (t-SNE) tool [28]. Figure 14 shows the eight selected features separate the network traffic into distinct and tightly packed clusters. Normal traffic converges into dense central core while blackhole, grayhole, flooding and scheduling attacks are pushed into cleanly separated peripheries. Such clean separation indicates why even a basic decision tree can classify the traffic with such high accuracy and low latency: the eight features form distinct, well-separated clusters in the projected space without requiring extensive non-linear deep learning transformations.

### 6.16. Targeted Adversarial Evasion and Defense Implications

To stress test FlowerSec-IDS against a realistic and intelligent adversary, we launched the Hop-Skip-Jump black-box attack [29] against 100 targeted malicious samples. This decision-based attack [30] repeatedly queries the model’s labels and walks along the decision boundary to find the cheapest perturbation that flips an attack into the normal class. The attack succeeded in evading detection in 80 of the 100 cases, with a median L2 perturbation distance of 2.764 and a mean of 3.888.

Analyzing the successful evasions reveals two critical nuances. First, the vulnerability is concentrated in the packet-dropping attacks: grayhole attacks accounted for 40 successful evasions and blackhole for 30, while flooding produced zero successful evasions. The volumetric signature of a flooding attack is essentially impossible to disguise without stopping the flood itself, meaning the most disruptive attack class remains fully detectable even under a targeted adversary. Second, the perturbations required for evasion are not free. A median L2 distance of 2.764 in the scaled feature space forces the attacker to substantially alter their traffic behavior, which directly degrades the effectiveness of the underlying attack.

To test whether adversarial training could mitigate this, we generated 181 Hop-Skip-Jump adversarial examples and added them to the training pool with their true labels [31]. The hardened model preserved its clean-set performance (Macro F1 decreased only marginally from 0.9494 to 0.9470). However, when re-evaluated against a fresh batch of Hop-Skip-Jump attacks, the evasion rate only decreased from 82 to 76 out of 100. This aligns perfectly with established adversarial machine learning literature, which highlights the specific vulnerability of tree ensembles to boundary-searching attacks [32]. Because Hop-Skip-Jump is an adaptive attack, it simply queries the newly hardened boundary to find a new evasion path. This validates our architectural decision to pursue a layered defense: the supervised detector must work in tandem with the unsupervised AutoEncoder to catch statistically anomalous traffic that slips past the decision boundary.

### 6.17. Scope and Boundaries

It is important to explicitly define the boundaries of the current validation. This paper rigorously validates the node-level AI engine (detection accuracy, inference latency, energy per packet, feature selection, zero-day detection, and adversarial robustness). However, it does not empirically validate the network-layer routing protocols using a network simulator (e.g., NS-3). Metrics such as packet delivery ratio, end-to-end latency, routing control overhead, cluster-head load, and network lifetime under identical radio assumptions require a full-scale simulation comparing FlowerSec against LEACH and HEED baselines. We explicitly reserve this comprehensive network-layer evaluation for our immediate future work, ensuring that the current claims remain strictly bounded by the empirical evidence presented in Section 6.

## 7. Conclusions and Future Work

In this paper, we proposed a novel AI-enhanced WSN security framework, FlowerSec, with a new developed topology called “flower” that contains a central seed node and at most four leaf nodes. We established a mathematically developed power-efficient clustering system to naturally outline the security boundary for a following real-time IDS system.

The FlowerSec architecture provides a rigorously validated node-level foundation for a powerful IDS, demonstrating the feasibility of deploying a lightweight, SHAP-optimized LightGBM model on the seed node for real-time intrusion detection. Experiment with WSN-DS showed the FlowerSec IDS model still maintained a Macro F1 score of 0.9494 on WSN-DS with only eight key features while having inference latency as low as 0.0066 ms and energy costs no more than 1.74 μJ.

Cross-dataset experiment with SensorNetGuard resulted in a Macro F1-score 0.9973, again proving the applicability of FlowerSec to WSNs. Most importantly, the leave-one-attack-class-out simulation showed that supervised learning alone was practically blind to unseen flooding traffic, detecting only 0.8% of it, while the unsupervised engine recovered 96.4%. This establishes that real-world WSN security requires both a fast supervised detector for known attacks and an unsupervised detector for zero-day threats, and FlowerSec provides a practical, energy-efficient way to deploy both on resource-constrained hardware. One area mentioned earlier is thermal and voltage drop effect: all energy calculations here remained careful projections based on host-level profiling. To measure actual effects, we will need to field-test our deployments on the ESP32 (and other) μC platforms. Another area where greater clarity may be obtained is on federated convergence (if not FL training). Since tree ensembles cannot simply be weight-averaged, neural network models had to be used as a surrogate for our FL simulation. It is unclear where exact convergence properties of a federated LightGBM would tend. Lastly, the threshold used for detection in the AE method was based on a fixed value, determined from testing. There clearly will be some environments where normal traffic is non-stationary and it may be beneficial/necessary to dynamically recalibrate this threshold; for now, the solution is only suitable for zero-day anomalies, whereas dynamic tuning makes the AE useful, as with our supervised method, for day-to-day anomaly detection too. These issues neatly mark out the limitations of this project work and in doing so provide the roadmap for its future development. Whilst these findings clearly show the suitability of using advanced ML at the edge, it must be acknowledged that the presented work is only foundational. Moving forwards, to further develop the system into a more rounded solution, we will:

1. Deploy the described components as a layered defense system as per the identified roadmap from our adversarial analysis, adding the supervised system to the AutoEncoder in combination with multi-seed consensus.
2. Investigate federated boosting variants (e.g., FedBoost-style histogram aggregation) for LightGBM to replace the neural surrogate used in our federated learning simulation.
3. Begin deployment on physical ESP32 hardware platforms to obtain realistic energy measurements, validate the cycle-count projections, and model thermal and voltage-drop effects.
4. Implement dynamic threshold recalibration for the AutoEncoder on a sliding window of verified normal traffic to handle non-stationary environments.
5. Develop noise-aware retraining and mobility-aware recovery mechanisms to further harden the framework against environmental degradation and node movement.

## Figures and Tables

**Table 1:** Comparison with recent AI-based WSN security approaches.

Study	AI Technique	Dataset	Accuracy	Key Limitation	FlowerSec Difference
CREN-RLC [4]	Regression learning	Custom	94.38%	No IDS, no recovery	IDS + recovery built in
SEEF [10]	Flower pollination	Custom	N/A	Algorithmic flower only	Physical flower topology
Voting + PSO [8]	Ensemble voting	WSN-DS	99.99%	Black box, all 19 features	Explainable, 8 features
Lightweight XGBoost [9]	XGBoost	WSN-DS	99.73%	No architectural mapping	Mapped to seed hardware
**FlowerSec (Ours)**	**LightGBM + AE**	**WSN-DS + 3**	**98.84%**	**Simulation only**	**Dual-engine, zero-day capable**

**Table 2:** Conceptual feature correspondence across the investigated datasets. This table maps abstract security categories to native feature names in each domain; it does not claim semantic or numerical equivalence. Each dataset is processed independently with its own features, scaler, and SHAP selection.

Concept	WSN-DS	SensorNetGuard	NSL-KDD	CIC-IDS2017
Packet activity rate	Derived from SCH_S	Packet_Rate	count	Flow Duration + Total Fwd Packets
Transmitted data volume	Data_Sent_To_BS	Data_Throughput	src_bytes	Total Fwd Packet Length
Error/loss behavior	Derived drop ratio	Error_Rate, Packet_Drop_Rate	serror_rate	Fwd Packets Dropped
Neighbor/CH interaction	ADV_R, ADV_S	Implicit	srv_count, dst_host_count	Flow IAT
Energy/resource state	Expanded Energy	Battery_Level, Energy_Consumption_Rate	Not available	Not available

**Table 3:** Performance comparison of intrusion detection models on WSN-DS dataset.

Model	Features	Accuracy	Macro F1	Train Time (s)	Inference (ms)	Size (KB)
XGBoost (16 Features)	16	0.9943	0.9663	5.57	0.0034	1140.66
LightGBM (16 Features)	16	0.9943	0.9667	4.89	0.0065	1718.37
FlowerSec IDS (Top-8)	8	0.9884	0.9494	4.10	0.0066	1715.68

**Table 4:** Assumptions of the network lifetime simulation and their justification.

Assumption	Value	Justification
Battery	2000 mAh at 3.7 V (26,640 J)	Standard 18650 Li-ion cell used in WSN testbeds
Baseline sensing/routing	50 J/day	First-order radio model, 100 packets/s, short-range TX
ML inference	1.74 μJ/packet	Host-profiled latency mapped to ESP32 active power
Layer activation	100%/15%/1%	Always-on, on-demand, and sleeping duty policies

**Table 5:** Zero-day AutoEncoder performance metrics (leave-one-attack-class-out).

Unseen Attack Class	Recall	Precision	F1-Score	FPR
Flooding	96.4%	94.1%	95.2%	5.0%
Grayhole	89.9%	88.5%	89.2%	5.0%
TDMA	93.4%	91.2%	92.3%	5.0%
Blackhole	100.0%	95.8%	97.9%	5.0%

**Table 6:** Comparison with state-of-the-art WSN intrusion detection methods on WSN-DS.

Study/Method	Features	Accuracy	Macro F1	Inference Time	Key Distinction/Limitation
Jaradat et al. (Voting + PSO) [8]	19 (All)	99.99%	≈98.5%	Not Reported	High computational overhead; lacks explainability.
Lightweight XGBoost [9]	19 (All)	99.73%	≈97.0%	Reduced by 68%	No architectural mapping to WSN topology.
FlowerSec IDS (Ours)	8 (SHAP)	98.84%	94.94%	0.0066 ms	Explainable, 50% feature reduction, mapped to seed-node architecture.

## Data Availability

Publicly available datasets were analyzed in this study. The WSN-DS dataset can be found at https://www.kaggle.com/datasets, accessed on 18 September 2026. The NSL-KDD and CIC-IDS2017 datasets are also publicly available via their respective repositories.
